# ﻿Typifications of 42 Mesoamerican names in Gesneriaceae, illustrated with type specimens and field images

**DOI:** 10.3897/phytokeys.267.169832

**Published:** 2025-12-12

**Authors:** Fred R. Barrie, Laurence E. Skog, John L. Clark, Kimberly Hansen

**Affiliations:** 1 Missouri Botanical Garden, 4344 Shaw Blvd., St. Louis, Missouri 63110, USA Department of Life Sciences, The Field Museum Chicago United States of America; 2 Department of Life Sciences, The Field Museum, 1400 S. Lake Shore Drive, Chicago, Illinois 60605, USA Missouri Botanical Garden St. Louis United States of America; 3 Department of Botany, MRC-166, Smithsonian Institution, PO Box 37012, National Museum of Natural History, Washington, District of Columbia 20013, USA Smithsonian Institution, National Museum of Natural History Washington United States of America; 4 Marie Selby Botanical Gardens, 1534 Mound St., Sarasota, Florida 34236, USA Marie Selby Botanical Gardens Sarasota United States of America

**Keywords:** Gesnerieae, Gesnerioideae, Mesoamericana, nomenclature, taxonomy

## Abstract

In preparation for the Gesneriaceae treatment in Flora Mesoamericana vol. 5(1), we review the typification of names applied to Mesoamerican taxa and designate lectotypes or neotypes for 42 names accepted in our treatment. Many earlier typifications—particularly for names published by Hanstein and based on material once at B (Berlin) and destroyed during WWII—were incomplete or ineffective under the International Code of Nomenclature (Madrid Code). We correct these by making second-step lectotypifications where required, clarifying typification statements in the literature, and selecting replacement types when original material is missing. All taxa are illustrated by digitized type specimens and other photographic evidence such as field images from the authors or conspecific iNaturalist records. The resulting typifications stabilize nomenclature across the following genera for Mesoamericana: *Achimenes*, *Besleria*, *Cobananthus*, *Columnea*, *Drymonia*, *Gasteranthus*, *Moussonia*, *Niphaea*, *Rufodorsia*, *Rhynchoglossum*, *Smithiantha*, and *Solenophora*.

## Abstract (es)

En preparación para el tratamiento de Gesneriaceae en “Flora Mesoamericana” vol. 5(1), revisamos la tipificación de los nombres aplicados a los taxones mesoamericanos y designamos lectotipos o neotipos para 42 nombres aceptados en nuestro tratamiento. Muchas de las tipificaciones anteriores, fueron incompletas o ineficaces según el Código Internacional de Nomenclatura (Código de Madrid),—particularmente aquellas publicadas por Hanstein y basadas en material que originalmente se encontraba en B (Berlín), destruido durante la Segunda Guerra Mundial . Para corregir estas situaciones, efectuamos designaciones lectotípicas aclaratorias cuando fue necesario, precisando declaraciones de tipificación previa y seleccionando tipos de reemplazo, en los casos en que el material originalestuviera ausente. Todos los taxones están ilustrados con especímenes tipo digitalizados y otras evidencias fotográficas, tales como imágenes de campo de los autores o registros de iNaturalist de ejemplares que pertenecen a la misma especie. Las tipificaciones resultantes estabilizan la nomenclatura de los siguientes géneros para Mesoamérica: *Achimenes*, *Besleria*, *Cobananthus*, *Columnea*, *Drymonia*, *Gasteranthus*, *Moussonia*, *Niphaea*, *Rufodorsia*, *Rhynchoglossum*, *Smithiantha* y *Solenophora*.

## ﻿Introduction

In preparation of the treatment of Gesneriaceae for Volume 5(1) of Flora Mesoamericana (Ulloa Ulloa et al., eds., in prep.), we discovered that the names of several species require lectotypification or neotypification. As all type specimens of Gesneriaceae at the Berlin herbarium (B), were lost during World War II, especially the types of names published by Hanstein (1865), previous authors have designated lectotypes or neotypes for the names of many species of Mesoamerican gesneriads. However, some of these designations do not meet all the requirements of the Madrid Code (Turland et al., eds., 2025) and their typification statements are either ineffective or incomplete. For example, in designating a lectotype or neotype, some authors cite only the collector and collection number without indicating the herbarium in which the type resides. In other cases, when multiple duplicates exist within a cited herbarium, they fail to specify which duplicate constitutes the lectotype or neotype. Article 9.17 of the Madrid Code provides the remedy for such cases of partial typification, i.e., the designation of a single specimen from among the gathering cited, as a second-step typification. Examples in this paper include the typification statements of Gibson (1974), who, in her treatment of the family for the Flora of Guatemala, often cited gatherings as “type” without specifying a single sheet or an institution. These are ‘first-step’ typification statements; the ‘second-step’ is taken here.

Occasionally, herbarium specimens have been annotated indicating “lectotype” or “holotype,” but when such designations have not been published, they are ineffective.

Here we designate lectotypes or neotypes for 42 previously untypified, or currently ineffectively typified, names of species accepted in our treatment of Gesneriaceae for Vol. 5 of Flora Mesoamerica, a multi-volume flora providing descriptions and keys for the plants occurring in Mesoamerica: Tabasco, Chiapas and the Yucatan in southern Mexico, and the countries of Central America. We cite the barcode number of the type, if available, or occasionally the sheet accession number. The numbers associated with the specimens are barcodes unless otherwise noted. We also include information about some typifications made by others to update and clarify their lecto- or neotypifications.

Particularly helpful has been the digitization of specimens, including images of types found in JStorPlants (www.plants.jstor.org), www.JACQ.org, and in individual digitization projects from many herbaria. Dowe et al. (2022) provided numerous new lectotypifications, in Gesneriaceae and other families, for the names of species described by Hanstein from Wendland’s Costa Rican collections, originally housed at Berlin, based on duplicate specimens at GOET and other herbaria. The contributions of Dowe et al. (2022) have been especially valuable.

The figures include images of the designated types along with field photographs from the wild or from horticultural or living research collections. The field images are mostly selected from the authors’ extensive fieldwork or carefully selected iNaturalist (https://www.inaturalist.org) observations that represent material conspecific with the typified name. Whenever possible, herbarium voucher specimens corresponding to the field images are cited. In cases where no voucher is available, the photographer and the iNaturalist observation are acknowledged.

## ﻿Taxonomic treatment

### ﻿*Achimenes skinneri* Lindl., J. Hort. Soc. London 2: 293, pl. 4, fig. 2. (1847). Lectotype, designated here, the colored illustration included with the original description, J. Hort. Soc. London, volume 2, plate 4, figure 2. (1847).

Fig. 1

**Figure 1.**
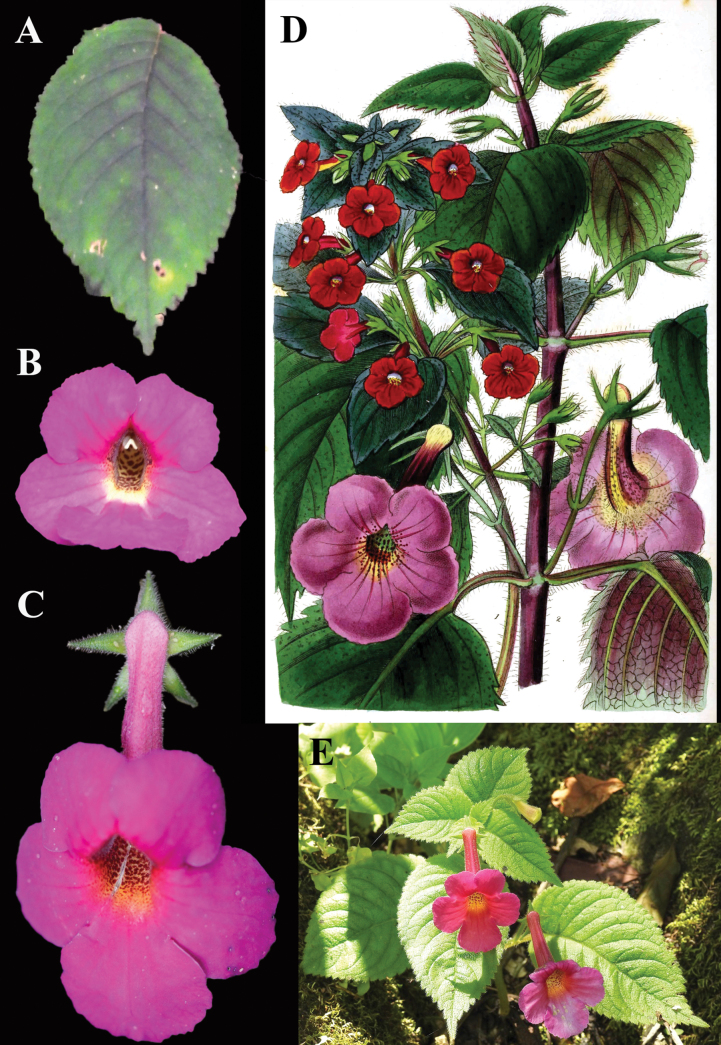
*Achimenes skinneri* Lindl. A. Upper leaf surface; B. Front view of corolla; C. Dorsal view of flower; D. Lithograph (plate 4, fig. 2) from Lindley (1847) with *A. skinneri* featured as the stem on the right (“2”) and the lower flowers; E. Habit (A. iNaturalist observation 201518021; B. iNaturalist observation 242545615; C. iNaturalist observation 16404629, E. iNaturalist observation 7611139). Photos (A) by Liliana Ramírez-Freire, (B, C, E) by Neptalí Ramírez Marcial.

**Comments.** In her revision of the genus *Achimenes*, Ramírez-Roa (1987: 130) cited the type as *Skinner s.n.* However, no original material traceable to either Skinner or Lindley (in Gordon 1850) has been located. Accordingly, the illustration accompanying the description has been selected as the lectotype (Fig. 1D).

### ﻿*Besleria barbensis* Hanst., Linnaea 34: 319 (1865). Neotype, designated here: Costa Rica, Heredia, Headwaters of Río Santo Domingo, ca. 3 km E of San Rafael de Vara Blanca, N slope of Volcán Barva, 16 Apr 1986, *Grayum 7189* (US! [US00460140]), isoneotype (MO! [MO acc. no. 04469658]).

Fig. 2

**Figure 2.**
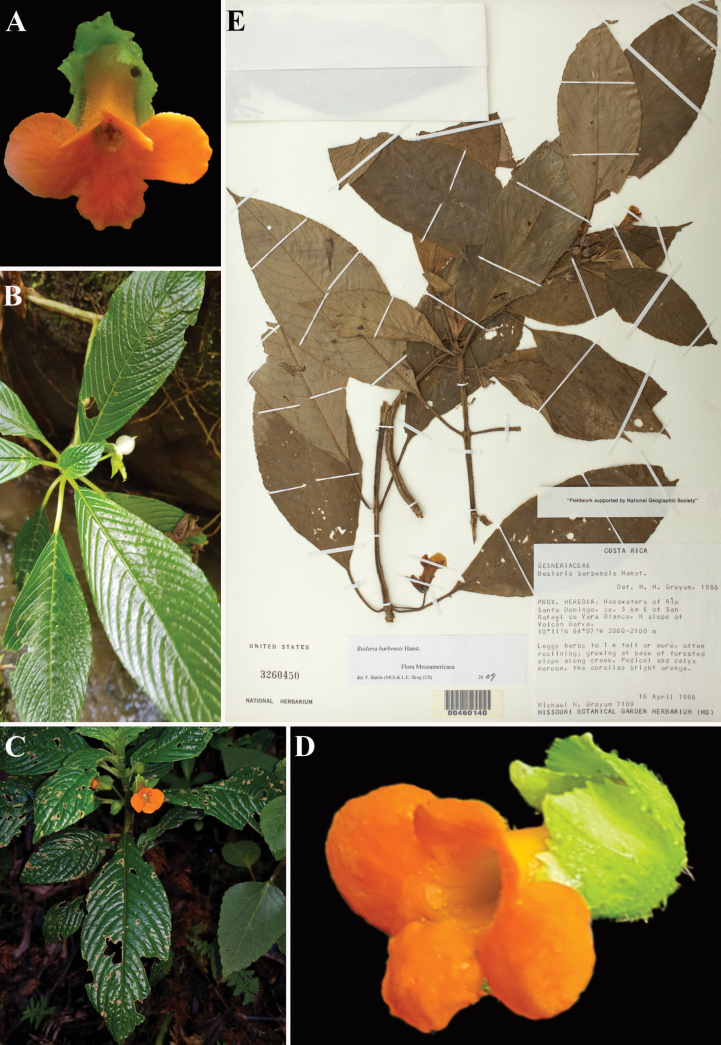
*Besleria barbensis* Hanst. A. Front view of flower; B, C. Habit; D. Lateral view of flower; E. Neotype from US of *Grayum 7189* [US00460140]. (A. iNaturalist observation 121592590; B. iNaturalist observation 61834131; C. iNaturalist observation 61834134; D. iNaturalist observation 66863407). Photos (A) by Donovan B. Drummey, (B–D) by Leonardo Álvarez-Alcázar.

**Comments.** The single specimen that Hanstein cited in the protologue, *Hoffmann no. 45* (B), was destroyed. No duplicates of the holotype have been located and no other original material for the name, uncited specimens or illustrations, is known. Therefore, a neotype (Fig. 2E) is designated here, collected, as was the holotype, on Volcán Barva.

### ﻿*Besleria columneoides* Hanst., Linnaea 34: 322 (1865). Lectotype, designated here: Costa Rica, San Miguel, *Wendland 762* (GOET (image)! [GOET003867]).

Fig. 3

**Figure 3.**
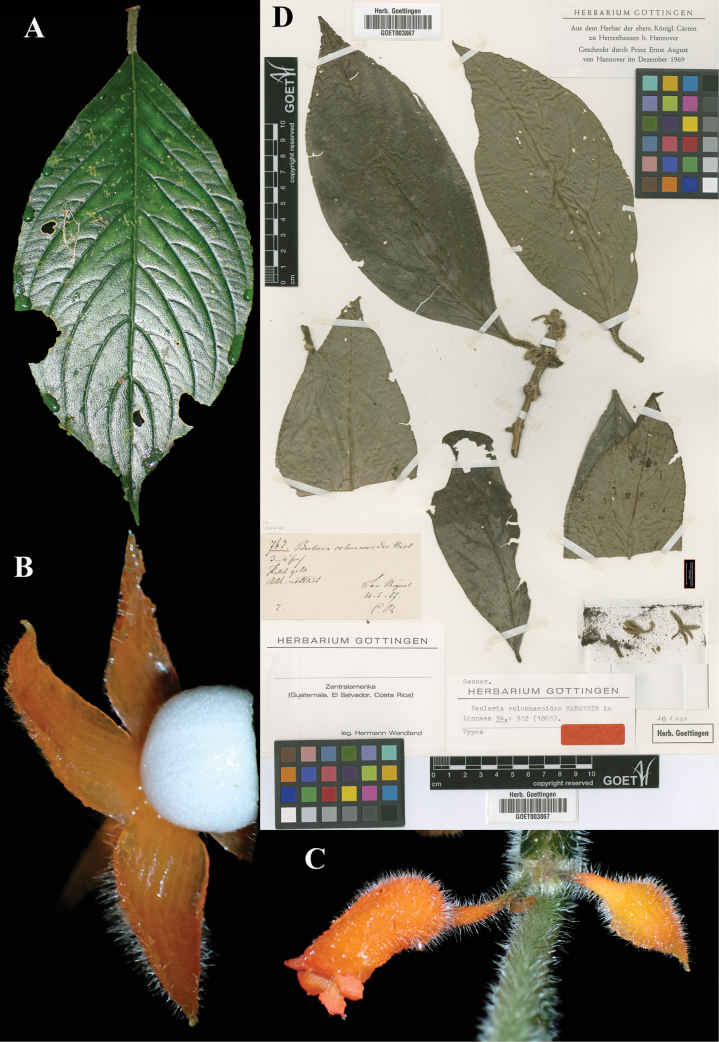
*Besleria columneoides* Hanst. A. Abaxial leaf surface; B. Mature globose berry; C. Mature flower; D. Lectotype from GOET of *Wendland 762* [GOET003867] (A–C. iNaturalist observation 199118732). Photos (A–C) by Dirk Mezger, (D) Specimen image reproduced with permission from the herbarium at the Universität Göttingen (GOET), Germany.

**Comments.** When he published *Besleria columneoides*, Hanstein cited a single gathering, *Wendland 762* (in “Plant. Wendl.”), but did not specify a herbarium, as he did for *B. barbensis*. *Wendland 762* included at least two specimens, one in Berlin, since destroyed, and another, now in the herbarium in Göttingen. In 2022, Dowe et al. (2022: p. 51) wrote that the lectotype had been designated by Morton (1938: 1151), in the “Flora of Costa Rica”. Morton, however, merely cited *Wendland 762* without identifying it as a type, nor does he, anywhere in his 1938 paper, indicate his intention to cite types. Consequently, his statement does not qualify as a lectotypification. In 1939, however, Morton did cite *Wendland 762* (B) as ‘Type’, effectively designating that specimen as the lectotype. Because the Berlin specimen was lost, the duplicate at GOET is designated here as the replacement lectotype (Fig. 3D).

### ﻿*Besleria flavovirens* Nees & Mart. Nova Acta Phys.-Med. Acad. Caes. Leop.-Car. Nat. Cur. 11: 49. 1823. Lectotype, designated by Skog and Feuillet (2008: 12): Brazil, *von Wied. s.n*. BR (image)! (BR691 181).

Fig. 4

**Figure 4.**
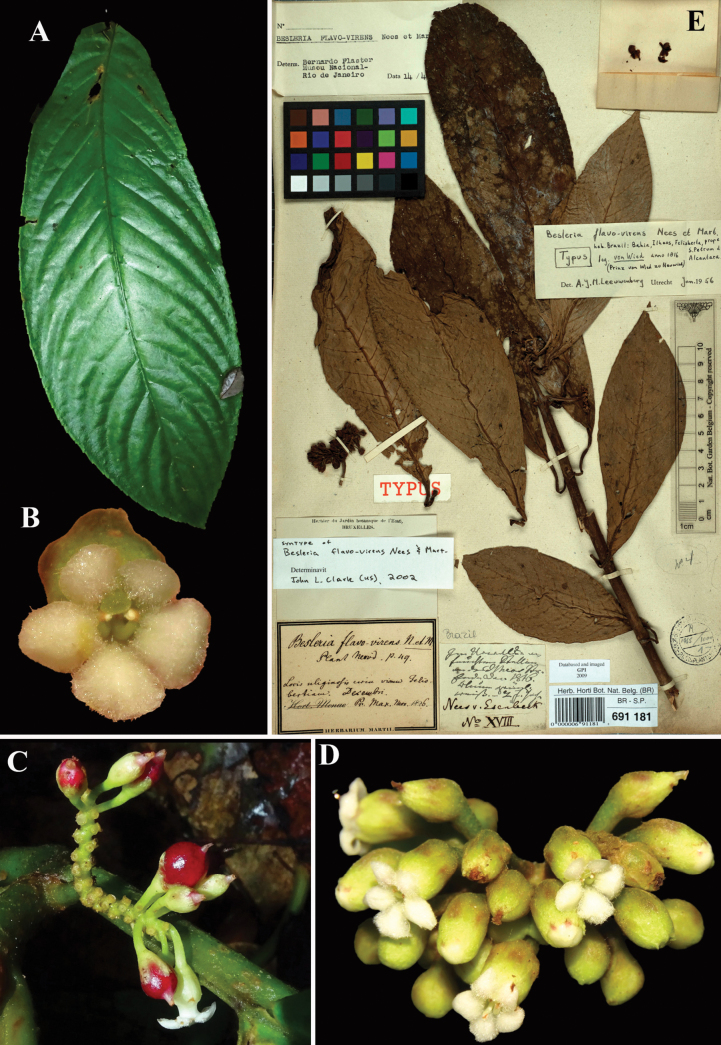
*Besleria flavovirens* Nees & Mart. A. Adaxial leaf surface; B. Front view of flower; C. Inflorescence with immature fruits; D. Inflorescence with mature flowers; E. Lectotype from BR of *von Wied s.n.* [BR000000691181] (A, C. iNaturalist observation 192925981, B, D. iNaturalist observation 9837872). Photos (A, C) by Olivier Fortune & Sabelle Delafosse, (B, D) by Rich Hoyer, (E) Specimen image reproduced with permission from the herbarium at the Meise Botanic Garden (BR), Belgium.

**Comments.** There are three syntypes of this species at BR. Skog and Feuillet (2008) designated as lectotype the specimen (Fig. 4E) labeled “typus” by Leeuwenberg (1958), which is the best preserved of the three syntypes.

### ﻿*Besleria princeps* Hanst., Linnaea 34: 317 (1865). Lectotype, (first step), Morton (1939: 465) *Wendland 1273*; second step (designated here): Costa Rica, *Wendland 1273* (GOET (image)! [GOET003869]).

Fig. 5

**Figure 5.**
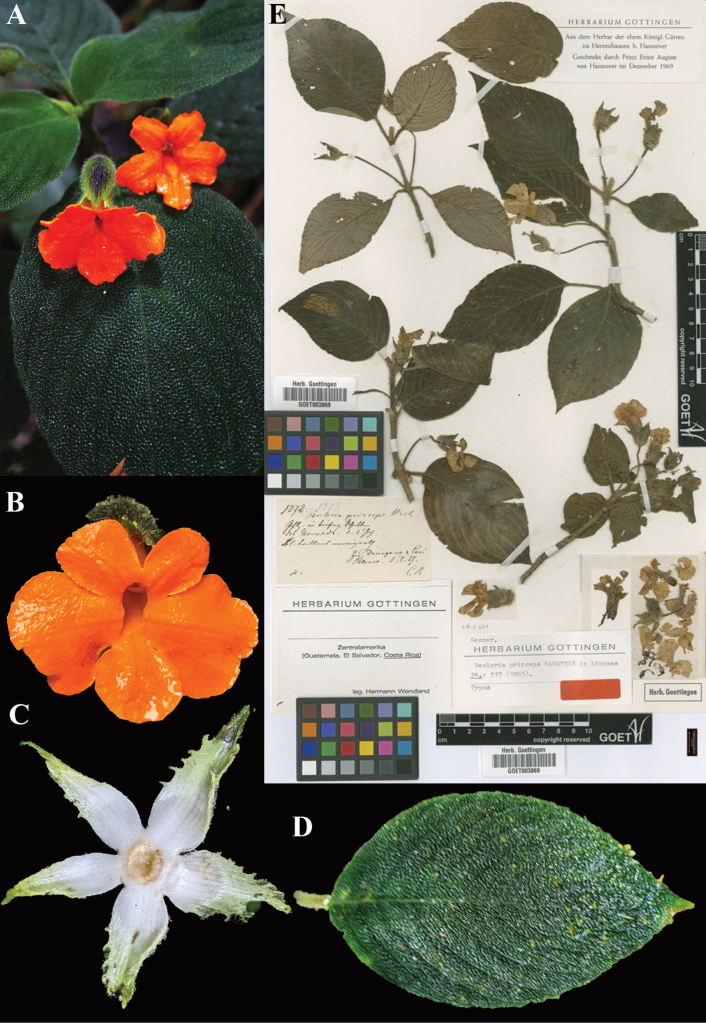
*Besleria princeps* Hanst. A. Habit; B. Front view of flower; C. Spreading calyx featuring annular nectary; D. Adaxial leaf surface; E. Lectotype from GOET of *Wendland 1273* [GOET003869] (A. from iNaturalist observation 254513781, B. from iNaturalist observation 176377119, C. iNaturalist observation 123867065, D. iNaturalist observation 179547056). Photos (A) by Markus Sonnberger, (B) by Isabella Han, (C) by Nick Seefeldt, (D) by Jacob Rehage, (E) Specimen image reproduced with permission from the herbarium at the Universität Göttingen (GOET), Germany.

**Comments.** As with *Besleria columneoides*, for *B. princeps* Hanstein cites a gathering, *Wendland 1273*, but does not specify a herbarium. Morton (1939: 465) cites the gathering as ‘Type’, but he also neglects to specify a herbarium. Presumably there was a specimen in Berlin, since destroyed. The only available original material that we have located is the duplicate at GOET (Fig. 5E), which we designate as lectotype.

### ﻿*Besleria triflora* (Oerst.) Hanst., Linnaea 34: 329 (1865). *Parabesleria triflora* Oerst., Centralamer. Gesner. 52, t. 6, figs. 1–8 (1858). Lectotype (first step), Morton (1939: 440) “Costa Rica, Cartago, Turrialba, *Oersted* (Co)”; second step (designated here): *Oersted 9285* (C (image)! [C10012718]), isolectotype (C (image)! [C10012719]).

Fig. 6

**Figure 6.**
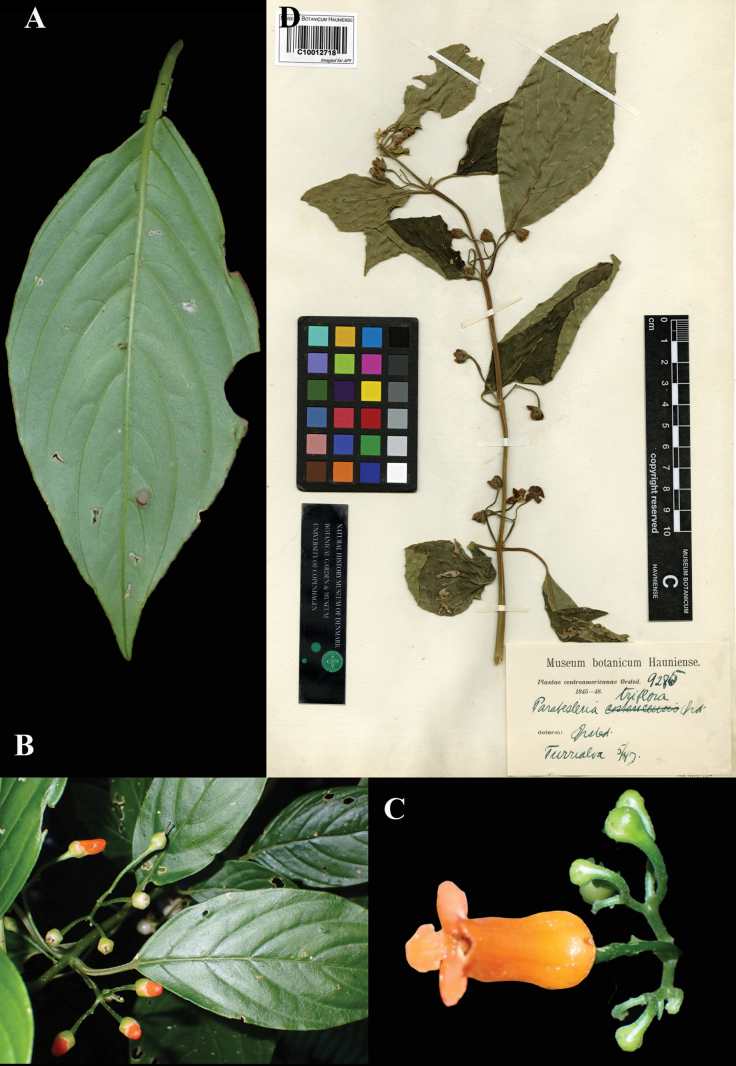
*Besleria triflora* (Oerst.) Hanst. A. Abaxial surface of leaf; B. Inflorescence and foliage; C. Mature flower; D. Lectotype from C of *Oersted 9285* [C10012718] (A–C. iNaturalist observation 200636039). Photos (A–C) by Dirk Mezger, (D) Specimen image reproduced with permission from the Natural History Museum Denmark.

**Comments.**Morton’s (1939) statement, “Type: Turrialba, Prov. Cartago, Costa Rica, Oersted (Co)” is acceptable as a first-step lectotypification. There are two specimens of *Oersted 9285* at Copenhagen (C), collected at “Turrialba”, bearing the basionym, *Parabesleria triflora*. We have selected what we believe to be the better specimen as lectotype (Fig. 6D).

### ﻿*Chrysothemis pulchella* (Donn ex Sims) Decne., Rev. Hortic., ser. 3. 3: 242 (1849); *Besleria pulchella* Donn ex Sims, *Bot. Mag.* 28, t. 1146 (1808). Lectotype designated by Leeuwenberg, 1958: 334: Cultivated, Aug. 1808, *Woodford s.n.* (BM (image)! [BM000617752]).

Fig. 7

**Figure 7.**
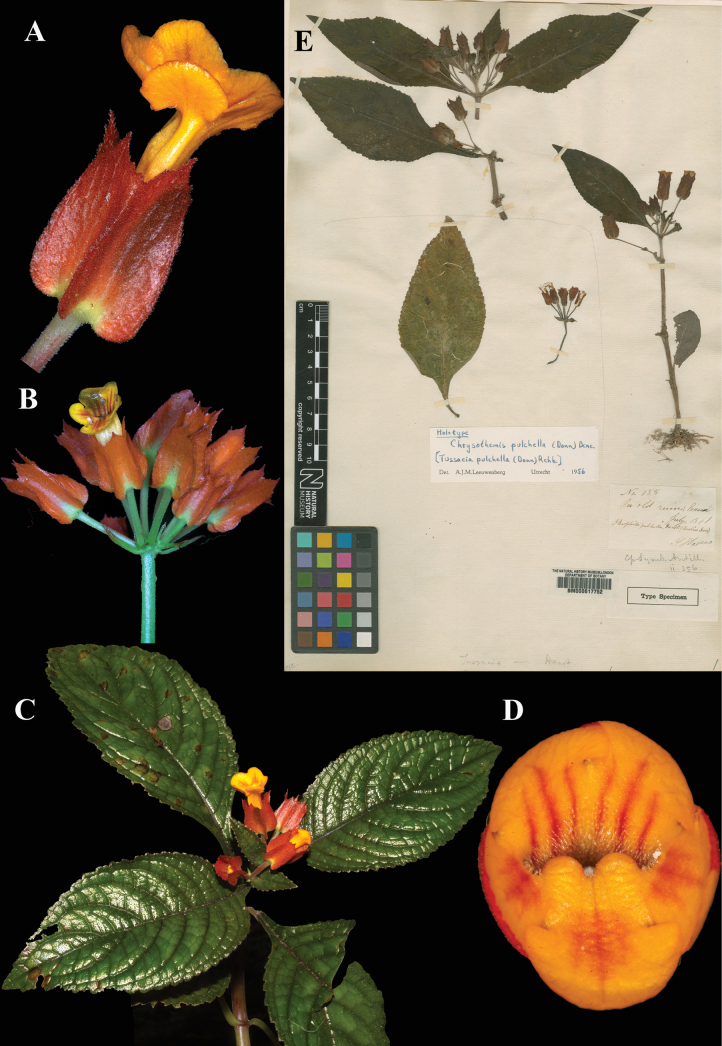
*Chrysothemis pulchella* (Donn ex Sims) Decne. A. Lateral view of flower; B. Inflorescence; C. Habit; D. Front view of corolla; E. Lectotype from BM of *Woodford s.n.* [BM000617752] (A, C, D. *J.L. Clark 18498*; B. *J.L. Clark 12746*). Photos (A–D) by John L. Clark, (E) Specimen image reproduced with kind permission from the Trustees, Natural History Museum, London (BM), United Kingdom.

**Comments.** Leeuwenberg cited this specimen as “Type”. There are, however, two elements of original material for Sims’ basionym, the Woodford specimen and the illustration published in the protologue, t. 1146. Therefore, Leeuwenberg’s citation of the Woodford specimen (Fig. 7E) is correctable to lectotype.

### ﻿*Cobananthus calochlamys* (Donn. Sm.) Wiehler, Selbyana 2: 94 (1977); *Alloplectus calochlamys* Donn. Smith, Bot. Gaz. 27: 437 (1899). Lectotype (first step), Gibson, 1974: 251; (second step), Wiehler, 1977: 94: Guatemala, Sacoyojú, May 1879, *Tuerckheim 456* (US! [US00126416]).

Fig. 8

**Figure 8.**
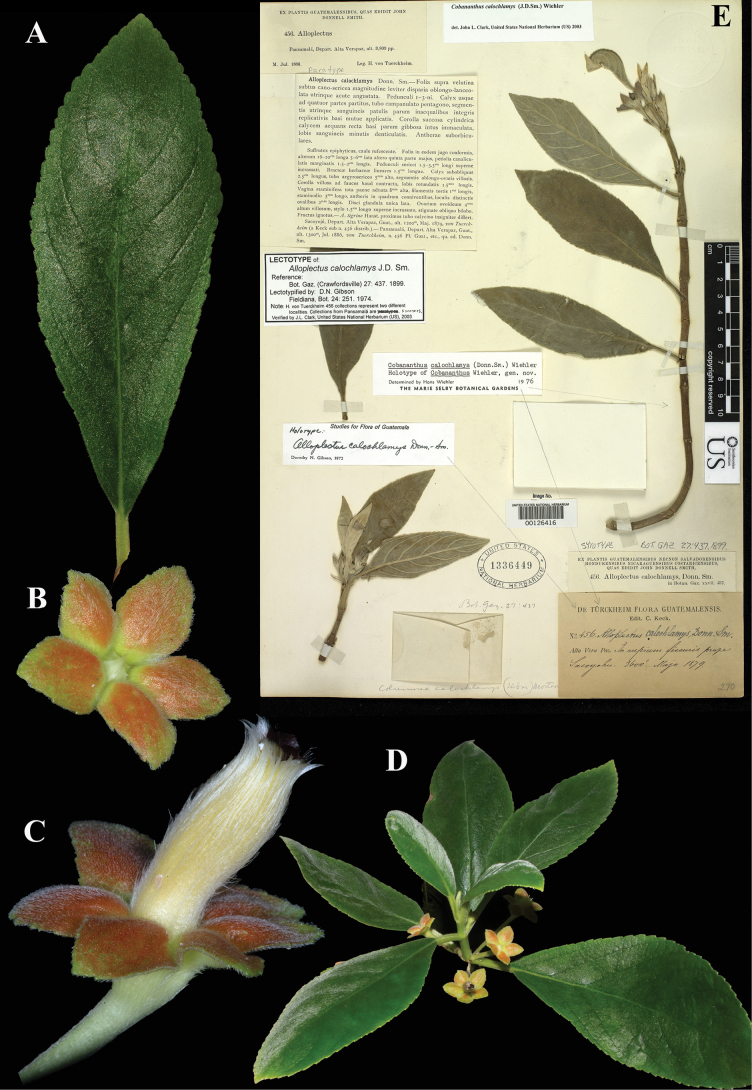
*Cobananthus calochlamys* (Donn. Sm.) Wiehler. A. Adaxial leaf surface; B. Calyx; C. Flower; D. Habit; E. Lectotype from US of *Tuerckheim 456* [US00126416] (A–D. *J.L. Clark 16364*). Photos (A–D) by John L. Clark.

**Comments.** John Donnell Smith cited two Tuerckheim gatherings in the protologue of *Alloplectus calochlamys*: ‘Sacoyojú’, collected in May, 1879, and ‘Pansamalá’, collected in June 1886. Both were distributed under the number *Tuerckheim 456*. Gibson (1974: 251) cited, as “Type”, the Sacoyojú gathering, but failed to indicate a herbarium. Her statement does, however, qualify as a first-step lectotypification, as it restricts the choice of lectotype to a specimen collected in 1879, excluding those collected in 1886. Three years later, Wiehler (1977: 94) took the second step, citing the US specimen (Fig. 8E) as “holotype”, an error correctable to lectotype.

### ﻿*Columnea calotricha* Donn. Sm., Bot. Gaz. 40: 9 (1905). Lectotype (first-step), designated by Gibson (1974: 253), *Tuerckheim 8542*; second-step (designated here): Guatemala, *Tuerckheim 8542* (US! [US00126463]).

Fig. 9

**Figure 9.**
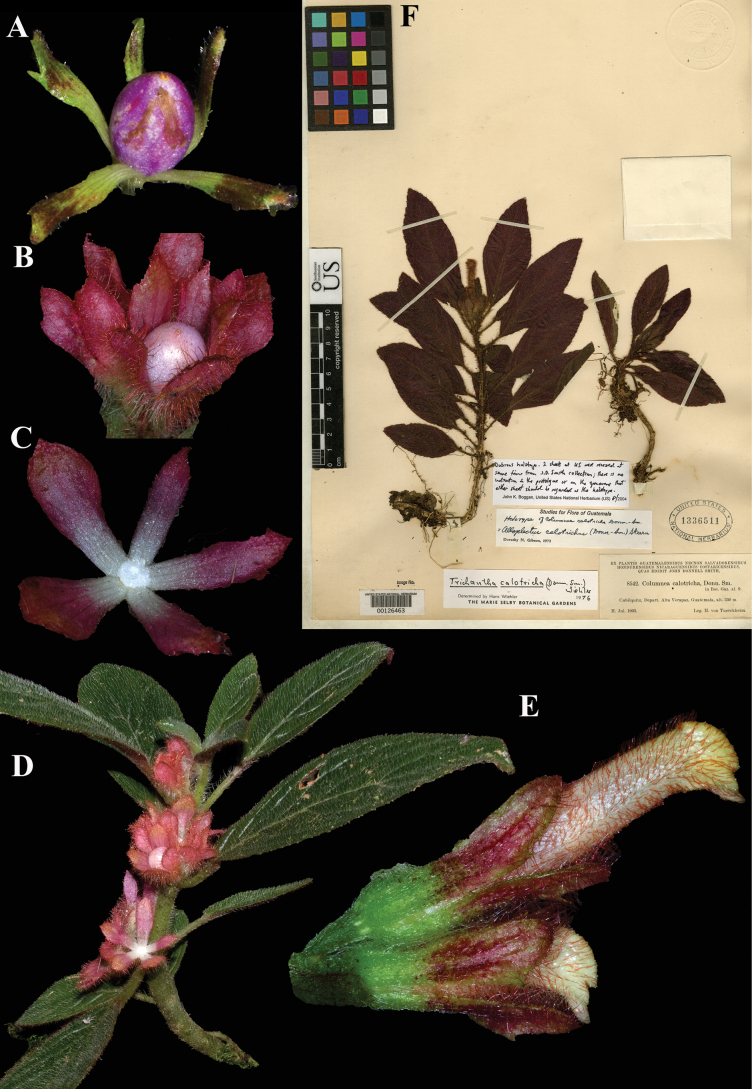
*Columnea calotricha* Donn. Sm. A, B. Fruit; C. Calyx; D. Habit; E. Lateral view of flower; F. Lectotype from US of *Tuerckheim 8542* [US00126463] (A, E. *J.L. Clark 14531*; B, C, D. *J.L. Clark & I. Pizarro 12629*). Photos (A–E) by John L. Clark.

**Comments.** The only original material for the name *C. calotricha* is the gathering cited by Donnell Smith in the protologue, *Tuerckheim 8542*. The John Donnell Smith collection at US includes two duplicates. Dorothy Nash Gibson, during her research for the Flora of Guatemala, annotated one sheet as holotype (Fig. 9F) and the second as isotype. We have selected the specimen that Gibson indicated as holotype to be the lectotype as it is the more complete specimen. The second Tuerckheim syntype becomes an isolectotype. In the Flora of Guatemala, Gibson treated this species under *Alloplectus*, and the name as the basionym of *A. calotrichus* (Donn. Sm.) Stearn.

### ﻿*Columnea cobana* Donn. Sm., Bot. Gaz. 57: 424 (1914). Lectotype (first-step), designated by Gibson (1974: 267); second-step (designated here): Guatemala, *Tuerckheim 2475* (US! [US00126470]).

Fig. 10

**Figure 10.**
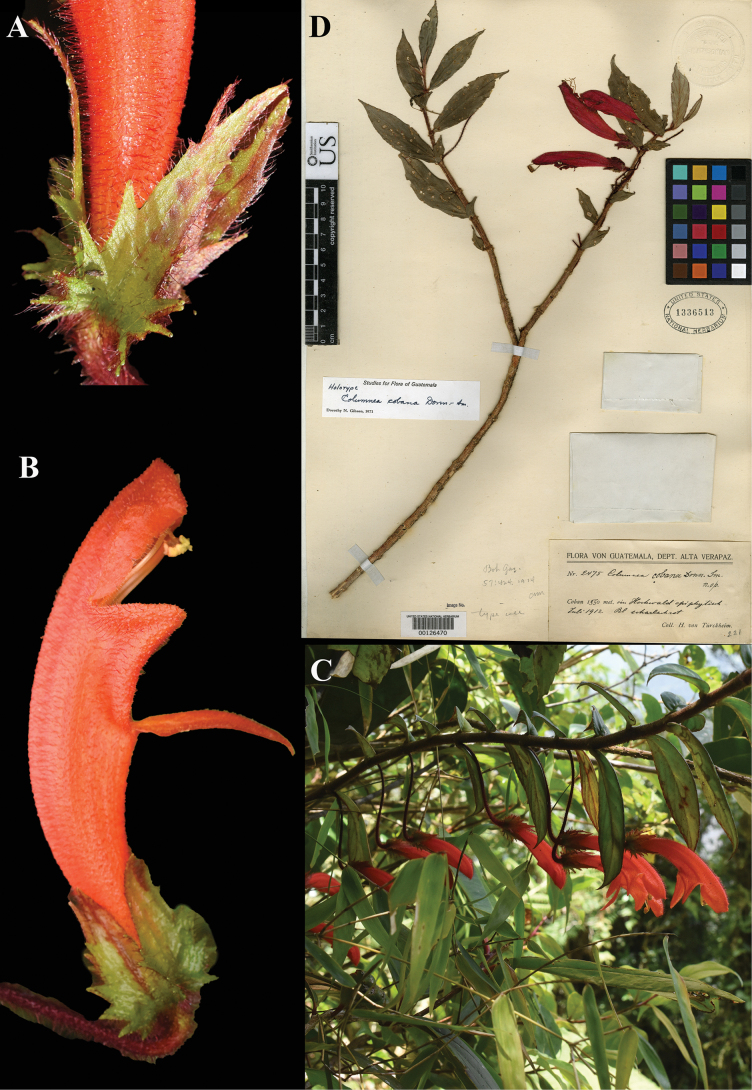
*Columnea cobana* Donn. Sm. A. Calyx; B. Flower; C. Habit; D. Lectotype from US of *Tuerckheim 2475* [US00126470] (A–F. iNaturalist observation 130310974). Photos (A–C) by Challen Willemsen.

**Comments.** Like the species above, there are two specimens (syntypes) at US with the same information (in different hands); like the species above, Donnell Smith cited the gathering in the protologue, but did not specify a type. Again, Gibson annotated one syntype specimen as holotype, and the other as isotype. The specimen labeled as holotype also has the words “type coll.” in the handwriting of Conrad V. Morton (1905–1972), specialist in Gesneriaceae at US. We have selected the specimen labeled as holotype by Gibson as lectotype (Fig. 10D); the other syntype is an isolectotype.

### ﻿*Columnea consanguinea* Hanst., Linnaea 34: 384 (1865). Lectotype, designated here: Costa Rica, *Wendland 509* (GOET (image)! [GOET003873]).

Fig. 11

**Figure 11.**
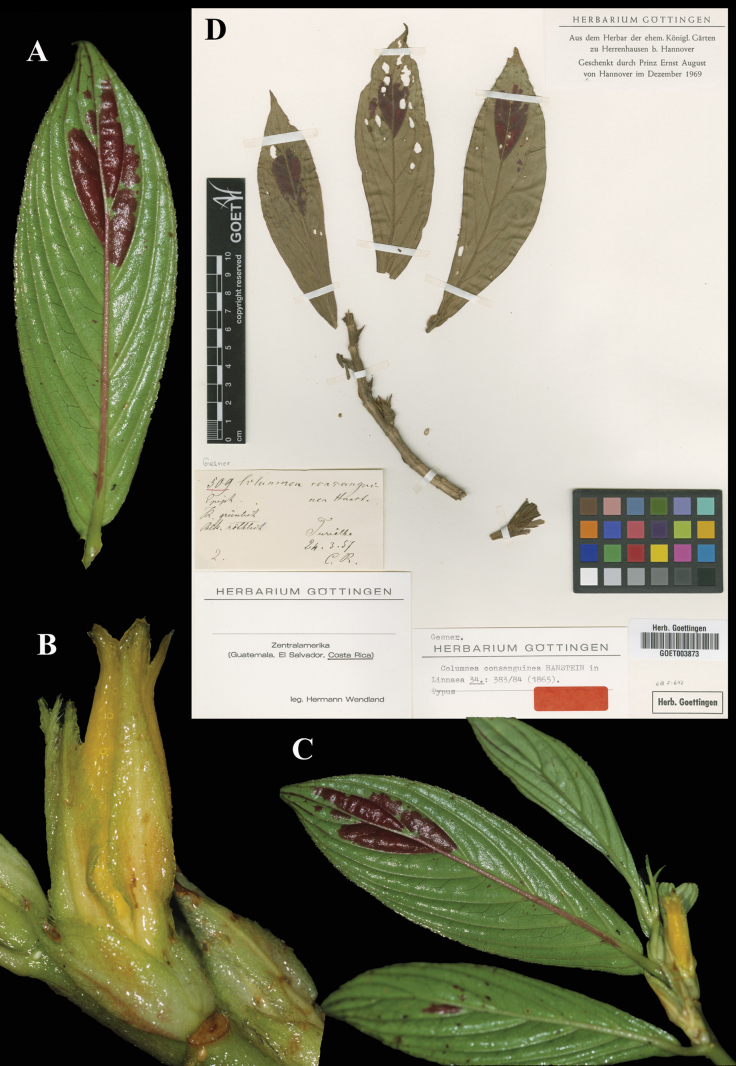
*Columnea consanguinea* Hanst. A. Abaxial leaf surface; B. Flower; C. Habit; D. Lectotype from GOET of *Wendland 509* [GOET003873] (A–C. *J.L. Clark 8705*). Photos (A–C) by John L. Clark, (D) Specimen image reproduced with permission from the herbarium at the Universität Göttingen (GOET), Germany.

**Comments.** This is another Hanstein name based on a single Wendland gathering, in this case *Wendland 509*. Presumably, Hanstein had a specimen at B, since destroyed. The duplicate at GOET is selected as lectotype (Fig. 11D). Dowe et al. (2022: 52) credits Morton (1971: 176–7) with designating the Wendland collection as lectotype. Morton, however, simply repeats Hanstein’s citation of the syntype gathering, “Turrialba, Costa Rica, *Wendland 509*”. Because he did not restrict the type citation in any way, Morton’s statement does not qualify as a lectotypification.

### ﻿*Columnea crassifolia* Brongn. ex Lem., Hort. Universel 5: 356 (1844). Neotype, designated here: Illustration in Lemaire, Fl. Serres Jard. Eur. 3(11): 286–286b (1847).

Fig. 12

**Figure 12.**
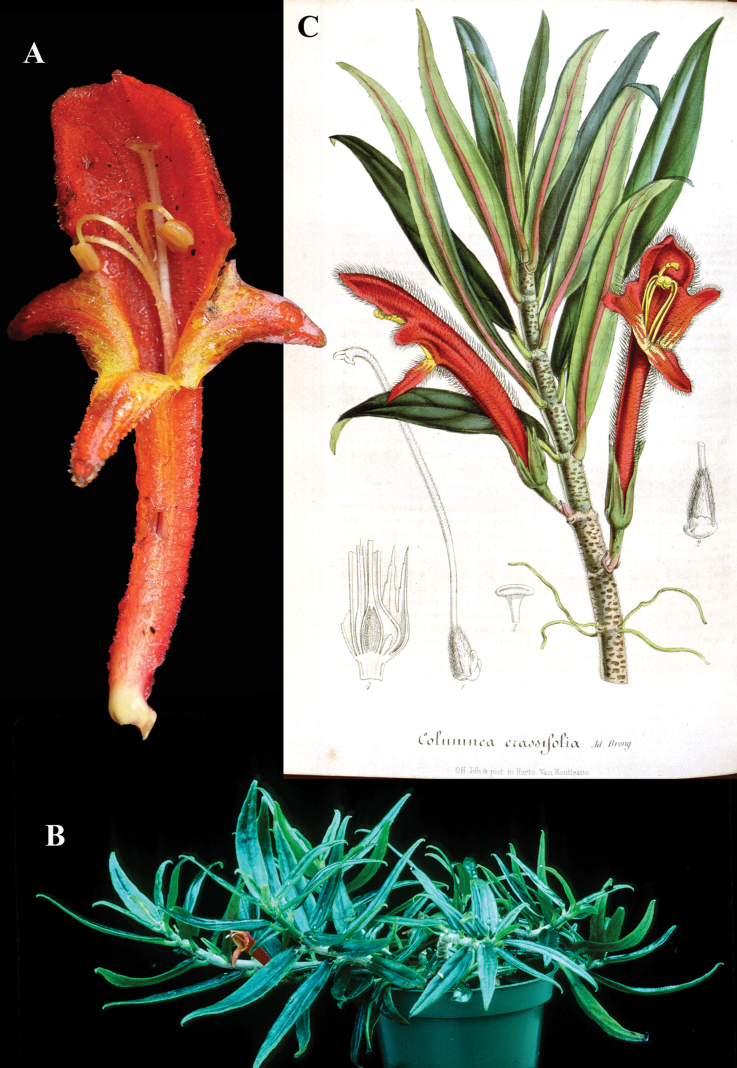
*Columnea crassifolia* Brongn. ex Lem. A. Flower; B. Habit; C. Lithograph from Lemaire (1847) (A. iNaturalist observation 272462314; B. United States Botany Research Greenhouses live collection #1995-023). Photos (A) by Jose Monzonsierra, (B) by Leslie Brothers.

**Comments.** No original collections are known for *Columnea crassifolia*, which was described from cultivation. The protologue does not include an illustration, but in a subsequent publication Lemaire (1847) provided an elegant illustration of *C. crassifolia*, in addition to a description. The 1847 illustration (Fig. 12C) is designated as the neotype.

### ﻿*Columnea erythrophaea* Decne. ex Houll., Rev. Hort. 1867: 172, t. 14 (1867). Lectotype, designated here: Mexico, Chiapas, Jitotol, *Linden 414* (K (image)! [K000641609]), isolectotype, (FI (image)! [FI009838I]).

Fig. 13

**Figure 13.**
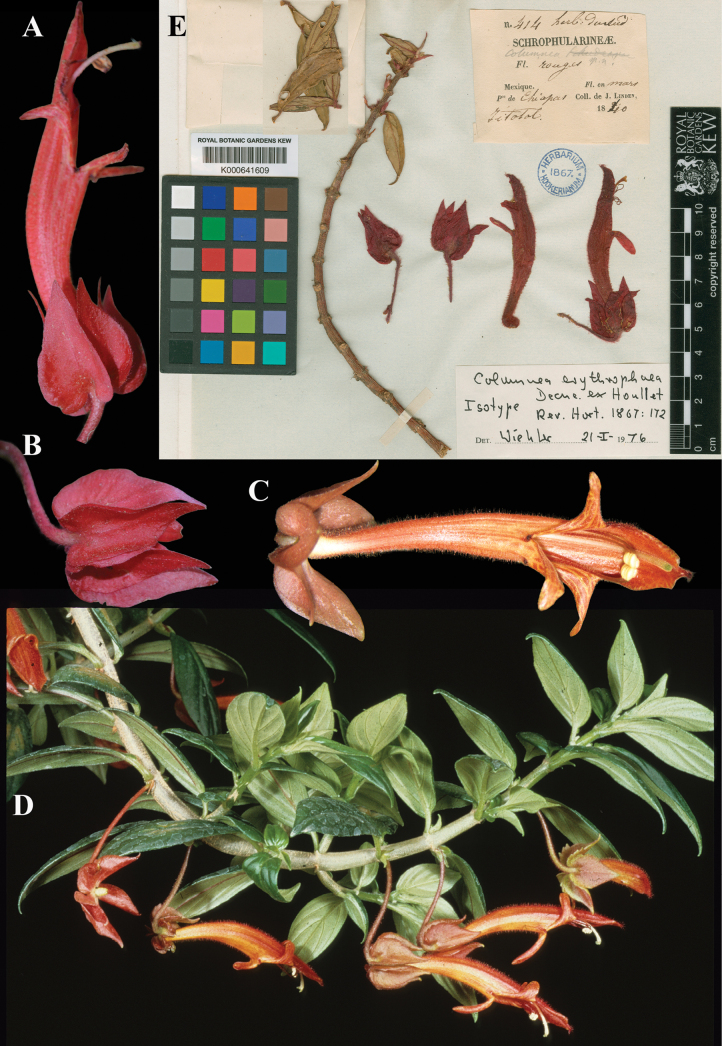
*Columnea erythrophaea* Decne. ex Houll. A, C. Lateral views of flower; B. Calyx; D. Habit; E. Lectotype from K of *Linden 414* [K000641609] (A, B. iNaturalist observation 205139723; C, D. *J.L. Clark 6723*). Photos (A, B) by Noel Alvarez, (C, D) by John L. Clark, (E) Specimen image reproduced with permission from the Board of Trustees of the RBG, Kew, United Kingdom.

**Comments.** We located two specimens that qualify as original material for this name: *Linden 414* at K and another at FI. In addition, there is the illustration published in the protologue. We designate the Kew specimen as the lectotype (Fig. 13E).

It is important to note that Linden did not uniquely number his collections. Other Linden gatherings, collected on different expeditions, on different dates, are also numbered 414. These collections, of other species, in various plant families, obviously are not original material for the name *C. erythrophea* and should not be confused with the specimens cited here.

### ﻿*Columnea flaccida* Seem., Bot. Voy. Herald 186 (1854). Lectotype, designated here: Panama, *Seemann 1163* (K (image!) [K000644060]).

Fig. 14

**Figure 14.**
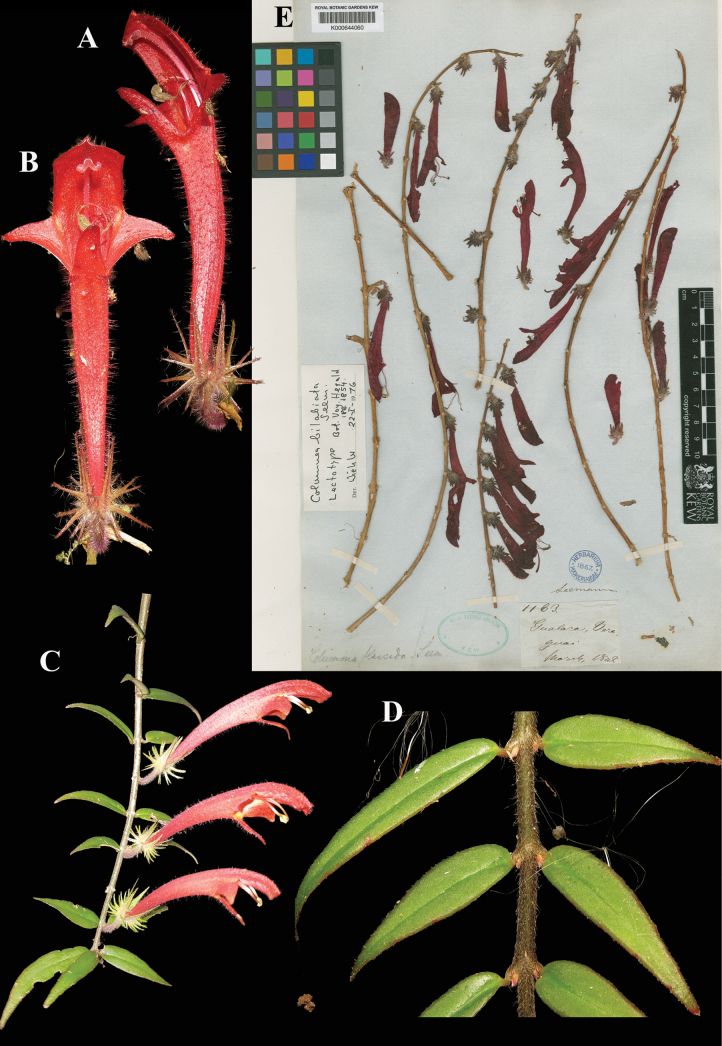
*Columnea flaccida* Seem. A, B. Lateral views of flower; C. Habit; D. Isophyllous foliage; E. Lectotype from K of *Seemann 1163* [K000644060] (A, B, D. iNaturalist observation 42365122; C. iNaturalist observation 42264739). Photos (A, B, D) by Rich Hoyer, (C) by Leonardo Álvarez-Alcázar, (E) Specimen image reproduced with permission from the Board of Trustees of the RBG, Kew.

**Comments.** There are two Seemann specimens labelled 1163, one at BM and one at K, that may be considered potential types. The entry for Seemann in TL-2 (Stafleu and Cowan 1985) says that “[T]he “study set” including the types of the *Bot. Voy. Herald* is at BM … with an important set also at K.” There is an interesting article dealing with the “study-set” of Seemann by Britten (1889). In the article the author describes that the types at BM include those of botanists other than Seemann who studied and wrote up treatments of other families, with none of those types at K. Britten further wrote: “With regard to the orders elaborated by Seemann himself, in so far as the plants are written up by him at the British Museum and Kew, they [the Seemann collections at BM and K] may be considered of equal weight.” The specimen bearing the collection number at K is more complete, includes the collection date, and the locality “Gualaca, Veraguas.” Seemann gives the locality in print as “Gualaca, Province of Veraguas.” Gualaca is a town in the current province of Chiriquí in Panama. The BM specimen has the words “Gualaca, Veraguas” in a different hand (and upside down and not on the Seemann collection number label), likely added later. Therefore, the specimen at K is designated as lectotype (Fig. 14E). Notable also is that the specimen at BM was annotated by Wiehler as an isotype of *Columnea flaccida*. The K specimen was annotated by Wiehler erroneously as lectotype of *Columnea bilabiata* Seemann [typified by *Seemann 1056*, and is from “Cape Corrientes, Darien“ according to the protologue, actually in the Dept. Chocó, Colombia]. Wiehler probably meant that he selected the specimen at K to be lectotype for *C. flaccida*.

### ﻿*Columnea glabra* Oerst., Centralamer. Gesner. 62, tab. XI, fig. 18–23 (1858). Lectotype, designated here: Costa Rica, Cartago, Candelaria, *Oersted 9287* (C (image)! [C10012728]), isolectotype (C (image)! [C10012727].

Fig. 15

**Figure 15.**
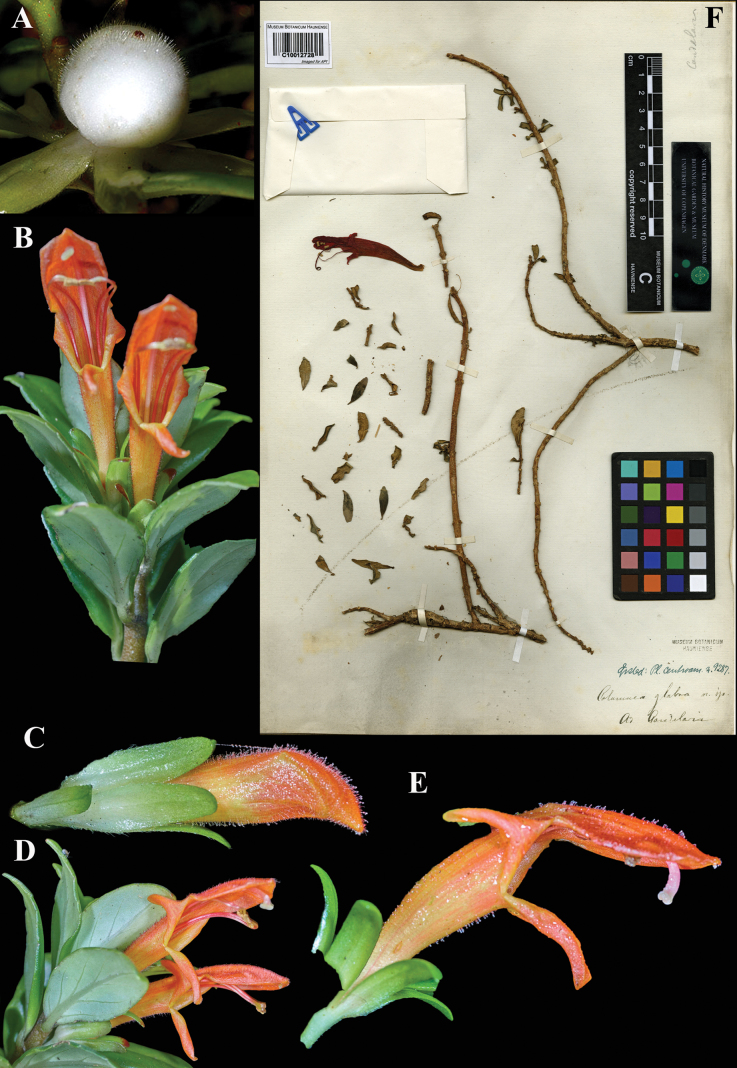
*Columnea glabra* Oerst. A. Mature globose berry; B. Habit; C. Lateral view of immature flower; D. Habit; E. Lateral view of mature flower; F. Lectotype from C of *Oersted 9287* [C10012728] (A–E. iNaturalist observation 97138506). Photos (A–E) by Sune Holt, (F) Specimen image reproduced with permission from the Natural History Museum Denmark.

**Comments.** There are two specimens of this number at C, and we selected what appears to be the more complete specimen (Fig. 15F) as lectotype.

### ﻿*Columnea gloriosa* Sprague, Bot. Mag. 137: tab. 8378 (1911). Lectotype, designated here: Cultivated at Kew in England from material collected in Costa Rica, *Anon. s.n.* (K (image)! [K000641610]).

Fig. 16

**Comments.**Sprague (1911) did not cite a specimen in the protologue. He described the plant as “purchased for the Kew collection from Messers. Haage & Schmidt.” Sprague’s description is clearly of a living plant, growing in the greenhouse.

**Figure 16.**
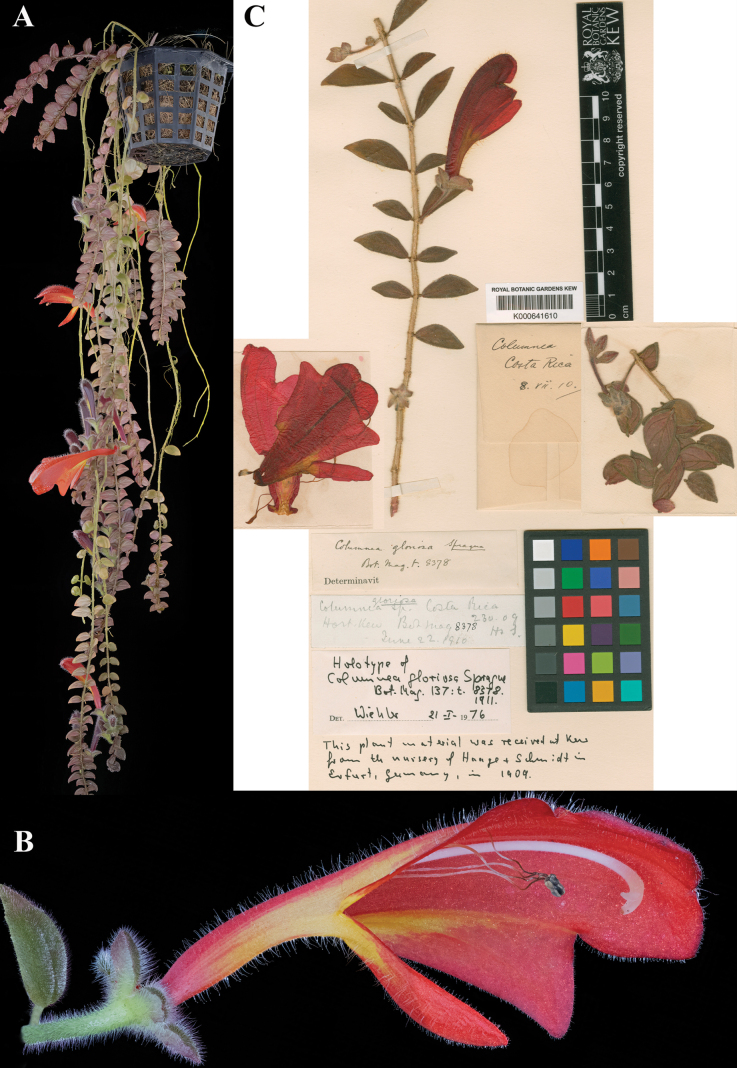
*Columnea gloriosa* Sprague. A. Habit; B. Lateral view of flower; C. Lectotype from K of *Anon. s.n.* [K000641610] (A, B. from *J.L. Clark & W. Collier 17645*). Photos by Wade L. Collier. (C) Specimen image reproduced with permission from the Board of Trustees of the RBG, Kew.

There are two elements of original material for *C. gloriosa*: the illustration published in the protologue. t. 8378; and a specimen collected anonymously. The specimen (Fig. 16C) bears a small label, in pencil, that reads “*Columnea gloriosa*, Costa Rica; Hort. Kew; Bot. Mag. 8378 [the number was added later, in ink]; H & S. June 22, 1910.” We interpret the label to indicate that the specimen was made from the original Haage & Schmidt plant in June 1910, and therefore known to Sprague prior to publication of the name. Hans Wiehler annotated the specimen as ‘holotype’ but never published his determination. We designate the specimen from K as the lectotype (Fig. 16C).

### ﻿*Columnea grata* C.V.Morton, Publ. Field Mus. Nat. Hist., Bot. Ser. 18: 1164 (1938). *Stenanthus heterophyllus* Oerst. ex Hanst., Linnaea 26: 208. (1854[1853]). *Columnea heterophylla* (Oerst. ex Hanst) Hanst., Linnaea 34: 390 (1865), non Roxburgh (1832: 97). Lectotype, designated here: Costa Rica, *Oersted 9288* (C (image)! [C10012729]); isolectotypes: C (image)! [C10012730], C (image)! [C10012731].

Fig. 17

**Figure 17.**
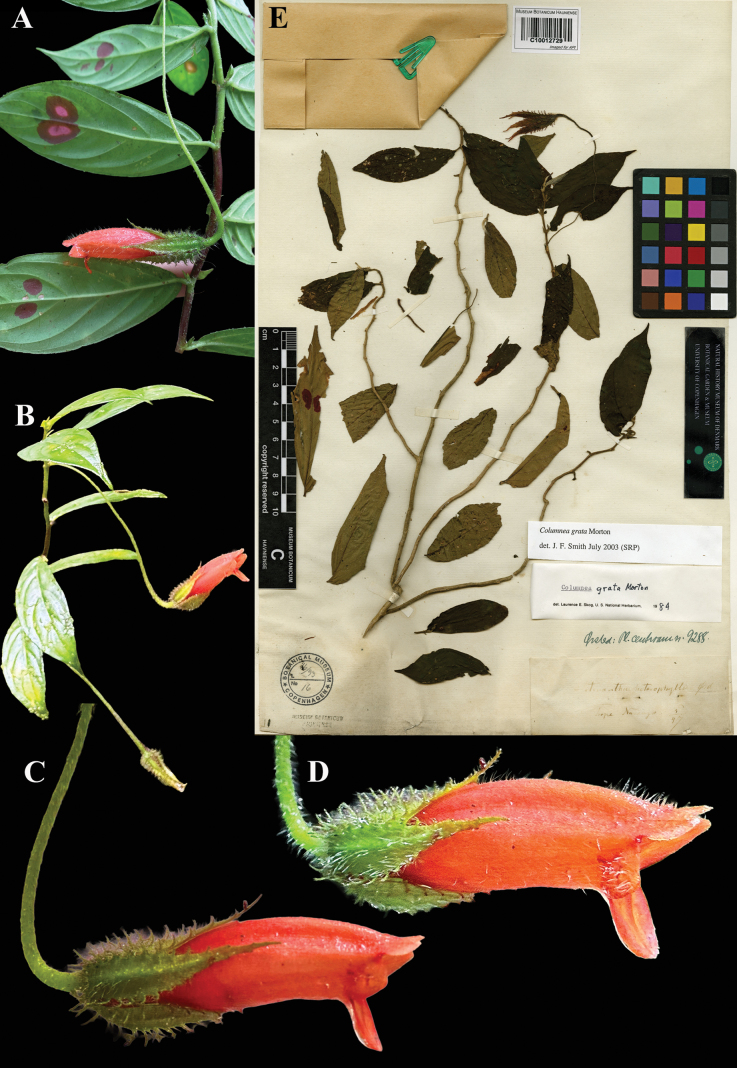
*Columnea grata* C.V. Morton. A, B. Habit; C, D. Lateral views of flowers; E. Lectotype from C of *Oersted 9288* [C10012729] (A. iNaturalist observation 232313386; B–D. iNaturalist observation 273426832). Photos (A) by Ariel Delgado, (B–D) by Irene Rosen, (E) Specimen image reproduced with permission from the Natural History Museum Denmark.

**Comments.** Morton published the replacement name *Columnea grata* for the illegitimate *C. heterophylla* Hanst. Hanstein ascribed the name *Stenanthus heterophyllus* to Oersted (“Oerst. in herb.”) and included a diagnostic illustration of the flower, but did not cite specimens. Neither Hanstein nor Oersted, in their publications of *Stenanthus heterophyllus* and later *Columnea heterophylla*, listed the Oersted specimen as “9288,” a number that was added later when the gathering was curated in Copenhagen; nor did Morton (1938) refer to this number in his renaming of the species. However, Oersted’s original label (see Fig. 17E), unnumbered but dated 5/47, gives Naranjo as the type locality, the locality cited by Oersted (1858) and Hanstein (1865), and again by Morton.

There are three duplicates of *Oersted 9288* in Copenhagen annotated as *Stenanthus heterophyllus* in Oersted’s handwriting. We selected the most complete specimen as the lectotype (Fig. 17E).

*Columnea grata* can be confused with *C. sanguinolenta* (Klotzsch ex Oerst.) Hanst. but is distinguished by the pedicels longer than the leaves and calyx fimbriae shorter than 4 mm.

### ﻿*Columnea hirta* Klotzsch & Hanst. Linnaea 34: 403 (1865). *Columnea hirsuta* Klotzsch ex Oerst. (1858), non *Columnea hirsuta*Swartz (1788). Neotype, designated here: Costa Rica, Cantón de Guápiles Los Angeles, San Miguel, Siguiendo el camino al Volcán Irazú, entre Río Blanco y Río Blanquito, 27 Feb 1990, *Chacón & Herrera 797* (US! [US00506126]); isoneotype: (MO! [MO acc. no. 401423]).

Fig. 18

**Figure 18.**
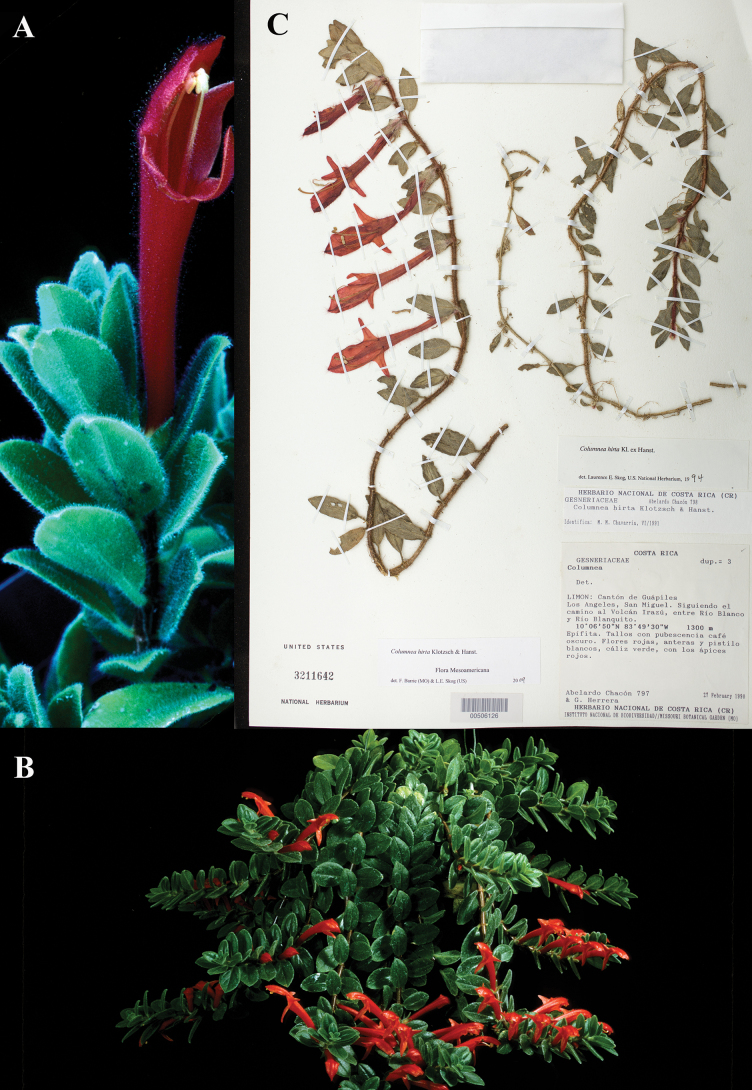
*Columnea hirta* Klotzsch & Hanst. A, B. Habit; C. Neotype from US of *Chacón & Herrera 797* [US00506126] (A, B. United States Botany Research Greenhouses live collection #1995-053). Photos (A, B) by Leslie Brothers.

**Comments.**Oersted (1858) cited an unnumbered Warszewicz collection from “Veragua” in the protologue. Hanstein (1865) cited *Warszewicz 32*, which may have been a reference to the same collection. No relevant Warszewicz specimen has been located, nor has *Wendland 564*, a second collection cited by Hanstein in 1865, which is not original material but might possibly have been an apt selection for the neotype. In its absence, a modern specimen (Fig. 18C) has been chosen.

### ﻿*Columnea lepidocaulis* (‘lepidocaula’) Hanst., Linnaea 34: 411 (1865). Lectotype, designated here: Costa Rica, *Valentini s.n.* (HAL (image)! [HAL0116066]).

Fig. 19

**Figure 19.**
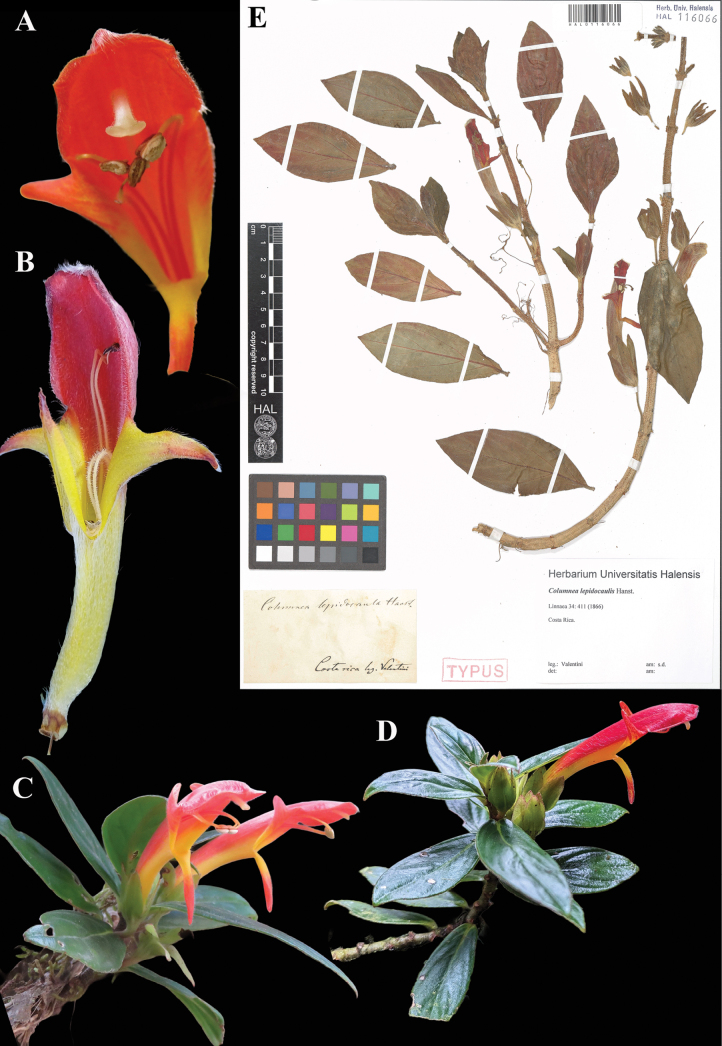
*Columnea lepidocaulis* Hanst. A, B. Mature flowers; C, D. Habit E. Lectotype from HAL of *Valentini s.n.* [HAL0116066] (A, C. iNaturalist observation 11181618; B. iNaturalist observation 215595684; D. iNaturalist observation 217789129). Photos (A, C) by Rich Hoyer, (B) by Andrey Loria, (D) by Amanda Bichel, (E) Specimen image reproduced with permission from Martin-Luther-Universität-Halle-Wittenberg herbarium (HAL), Germany.

**Comments.** Hanstein cited two gatherings in the protologue, *Valentini s.n.* and *Wendland 917*. A specimen of the former is at HAL; one of the latter is at GOET. Dowe et al. (2022) wrote that Morton (1938: 1165) had designated the Valentini specimen as lectotype. However, Morton did not indicate that the specimen was a type, nor is there a general statement in the publication that would indicate his intent to cite types. Therefore, his citation of ”Valentini” cannot be accepted as a type designation. The specimen is, however, an appropriate lectotype and is designated as such here (Fig. 19E).

### ﻿*Columnea nicaraguensis* Oerst., Centralamer. Gesner. 62 (1858). Lectotype, designated here: Nicaragua, *Oersted 9290* (C (image)! [C10012735]); isolectotype: (C (image)! [C10012736]).

Fig. 20

**Figure 20.**
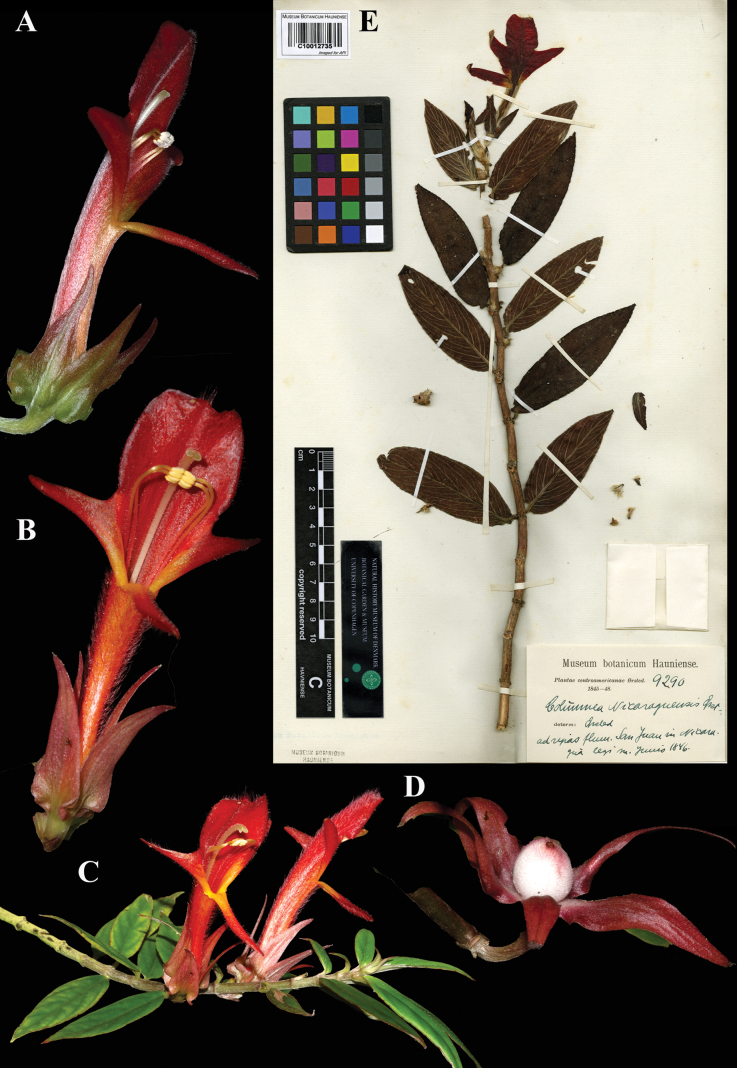
*Columnea nicaraguensis* Oerst. A, B. Mature flowers; C. Habit; D. Mature globose berry; E. Lectotype from C of *Oersted 9290* [C10012735] (A. *J.L. Clark 10643*; B, C. *J.L. Clark 16368*; D. *J.L. Clark 10643*). Photos (A–D) by John L. Clark, (E) Specimen image reproduced with permission from the Natural History Museum Denmark.

**Comments.** There are two specimens collected by Oersted at Copenhagen annotated as this species. We selected the more complete specimen as lectotype (Fig. 20E).

### ﻿*Columnea oerstediana* Klotzsch ex Oerst., Centralamer. Gesner. 61 (1858). Lectotype, designated by Leeuwenberg (1958: 388), as “holotype”: Costa Rica, *Oersted 9291* (C (image)! [C10012739]); isolectotypes (C (image)! [C10012738], C (image)! [C10012737], US! [US00126489], US! [US00223524]).

Fig. 21

**Figure 21.**
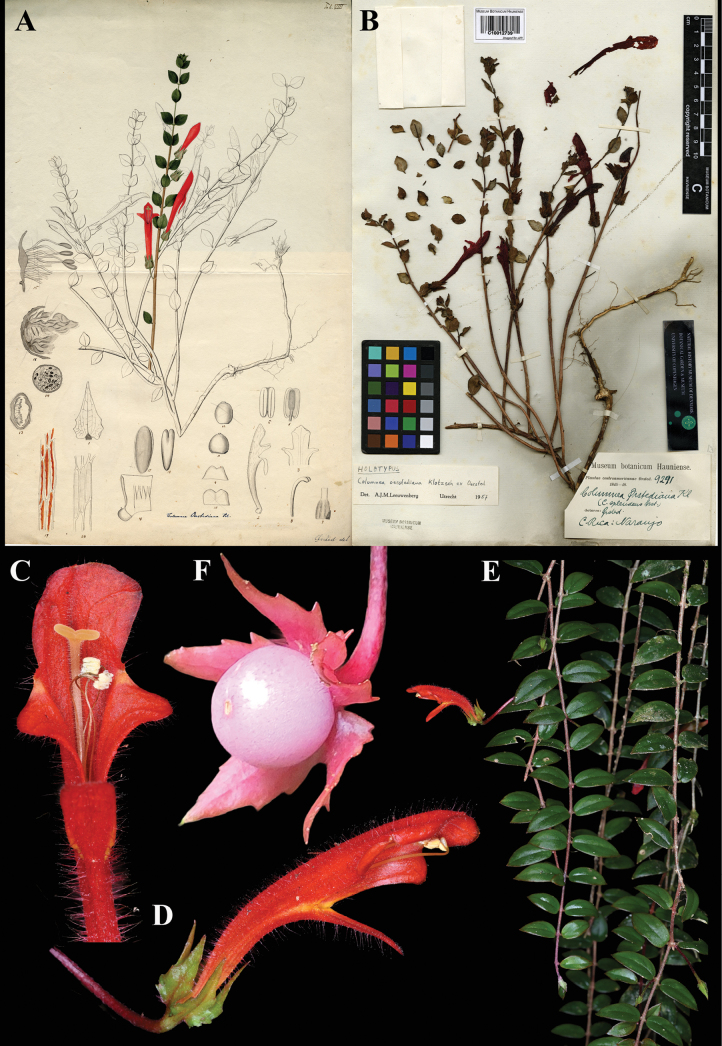
*Columnea oerstediana* Klotzsch ex Oerst. A. Tab.VIII from Oersted (1858); B. Lectotype from C of *Oersted 9291* [C10012739]; C, D. Mature flowers; E. Habit; F. Mature globose berry fruit (C–E. iNaturalist observation 192967144; F. iNaturalist observation 141547883). Photos (C–E) by Robin Heymans, (F) by ombeline_sculfort, (A, B) Specimen image reproduced with permission from the Natural History Museum Denmark.

**Comments.** There are three specimens at Copenhagen of *Oersted 9291* as well as two sheets of the same number at US. Leeuwenberg labeled one of the Copenhagen specimens as holotype (see Fig. 21B), an error correctable to lectotype, and the other two at C, as well as the specimens at US, as isotypes. He may have selected the sheet as holotype because it appears to resemble the plant illustrated by Oersted (1858) in his tab. VIII (Fig. 21A). One of the isotypes at C appears to have been annotated by Oersted, while the lectotype does not.

### ﻿*Columnea oxyphylla* Hanst., Linnaea 34: 405 (1865). Lectotype, designated here: Costa Rica, *Wendland 778* (GOET (image)! [GOET003877]).

Fig. 22

**Figure 22.**
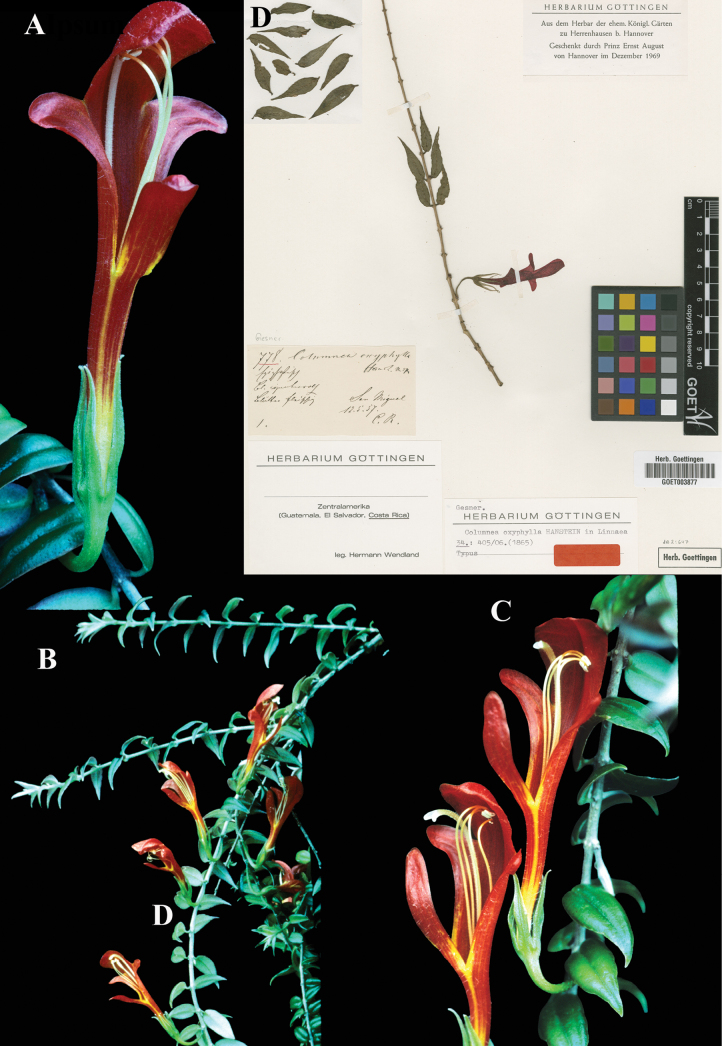
*Columnea oxyphylla* Hanst. A. Mature flower; B. Habit; C. Mature flower; D. Lectotype from GOET of *Wendland 778* [GOET003877] (A–C. from United States Botany Research Greenhouses live collection #1994-560). Photos (A–C) by Leslie Brothers, (D) Specimen image reproduced with permission from the herbarium at the Universität Göttingen (GOET), Germany.

**Comments.**Dowe et al. (2022) wrote that Morton (1938: 1167) had designated *Wendland 778*, the gathering cited by Hanstein, as lectotype. As noted above, however, Morton’s 1938 specimen citations are not acceptable as designations of lectotypes, as he neither called them types nor indicated a herbarium. As far as is known, the GOET specimen (Fig. 22D) is the only extant syntype.

### ﻿*Columnea purpurata* Hanst., Linnaea 34: 386 (1865). Lectotype, designated here: Costa Rica, *Wendland 548* (GOET (image)! [GOET003878]).

Fig. 23

**Figure 23.**
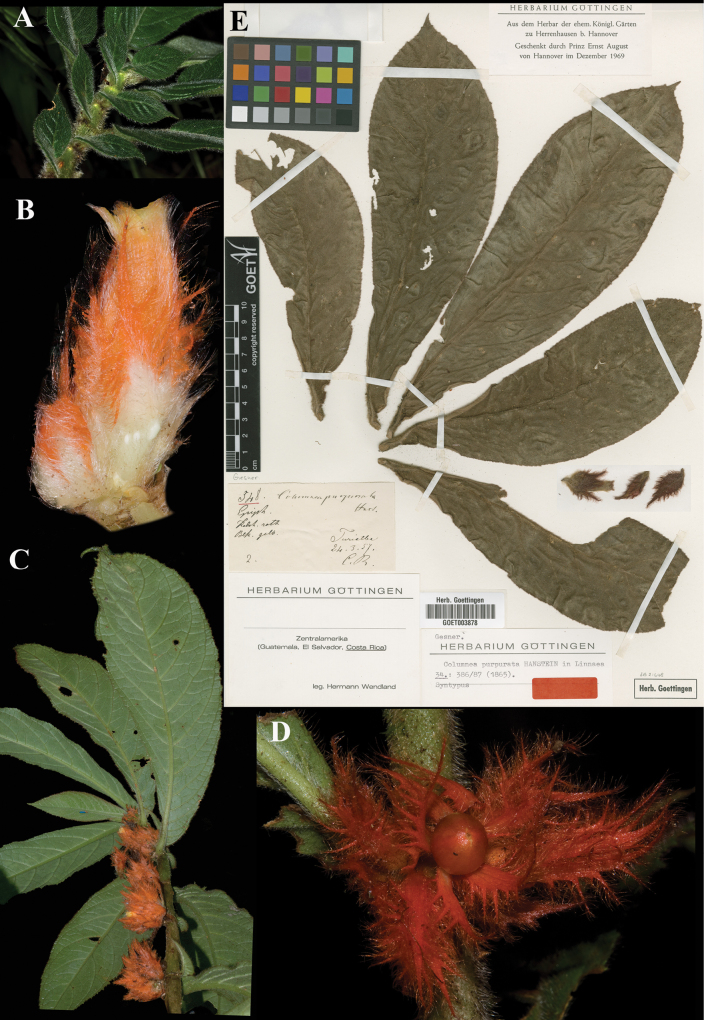
*Columnea purpurata* Hanst. A. Anisophyllous phyllotaxy; B. Mature flower; C. Dorsiventral habit; D. Mature globose berry; E. Lectotype from GOET of *Wendland 548* [GOET003878] (A. from *J.L. Clark et al. 12757*; B, C. *J.L. Clark & I. Pizarro 12604* D. *J.L. Clark & I. Pizarro 12671*). Photos (A–D) by John L. Clark, (E) Specimen image reproduced with permission from the herbarium at the Universität Göttingen (GOET), Germany.

**Comments.**Hanstein (1865: 386–7) listed three collections for *Columnea purpurata*: “*Wendland n.* 548; *Warszewicz 242 (6?)*; *Valentini*.” (specimens likely residing at B, and later destroyed), as well as citing “*C. purpurata* Hanst. in Plant. Wendl. n. 548.” Dowe et al. (2022: 52) wrote that Morton (1938: 1168) had designated *Wendland 548*, GOET (Fig. 23E) as the lectotype. As noted above, however, Morton’s 1938 specimen citations are not effective designations of types.

### ﻿*Columnea querceti* Oerst., Centralamer. Gesner. 59 (1858). Lectotype designated here: Costa Rica, *Oersted 9292* (C (image)! [C10012741]; isolectotype: C (image)! [C10012740]).

Fig. 24

**Figure 24.**
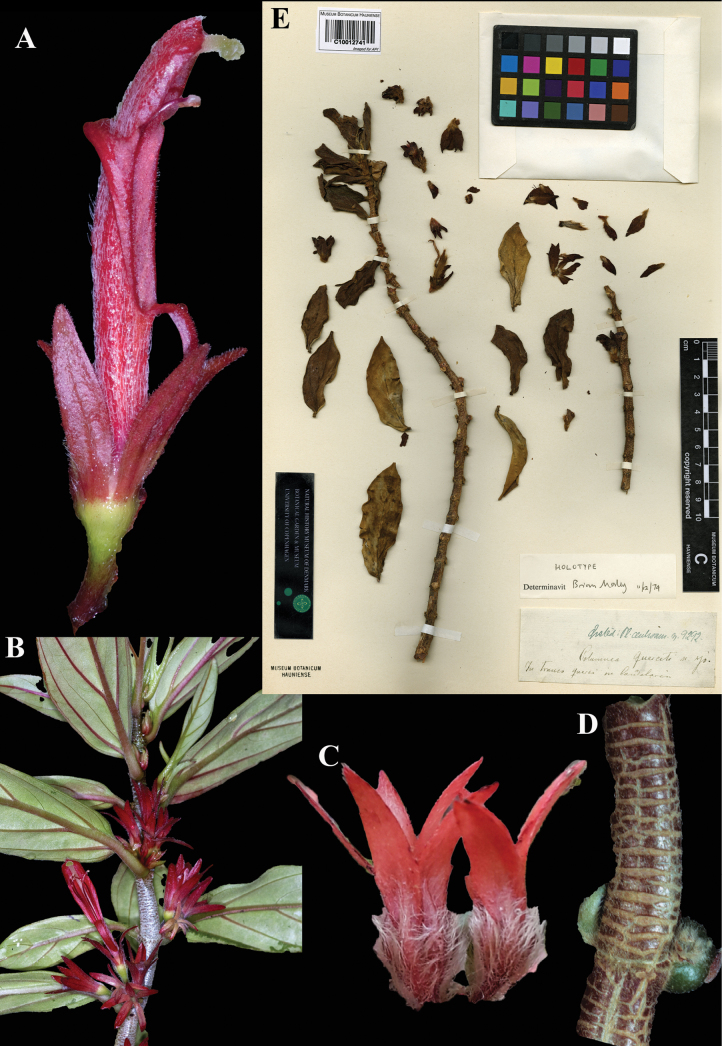
*Columnea querceti* Oerst. A. Mature flower; B. Habit; C. Mature calyces; D. Stem featuring peltate-like scales; E. Lectotype from C of *Oersted 9292* [C10012741] (A, B. *J.L. Clark 10047*; C. iNaturalist observation 5109522; D. Hans Wiehler live collection #G527). Photos (A, B) by John L. Clark, (C) by Steven Easley, (D) by Margaret H. Stone, (E) Specimen image reproduced with permission from the Natural History Museum Denmark.

**Comments.** There are two syntypes collected by Oersted in the Copenhagen herbarium. In 1974, Brian Morley annotated the lectotype (Fig. 24E) which he designated as ‘holotype’ but never stated so in a publication.

### ﻿*Columnea sanguinolenta* (Klotzsch ex Oerst.) Hanst., Linnaea 34: 389 (1865). *Stenanthus sanguinolentus* Klotzsch ex Oerst., *Centralamer. Gesner.* 49 (1858). Neotype, designated here: Costa Rica, Guanacaste, La Tejona, N of Tilaran, 600–700 m, 25 Jan 1926, *Standley & Valerio 45757* (US! [US00223799].

Fig. 25

**Figure 25.**
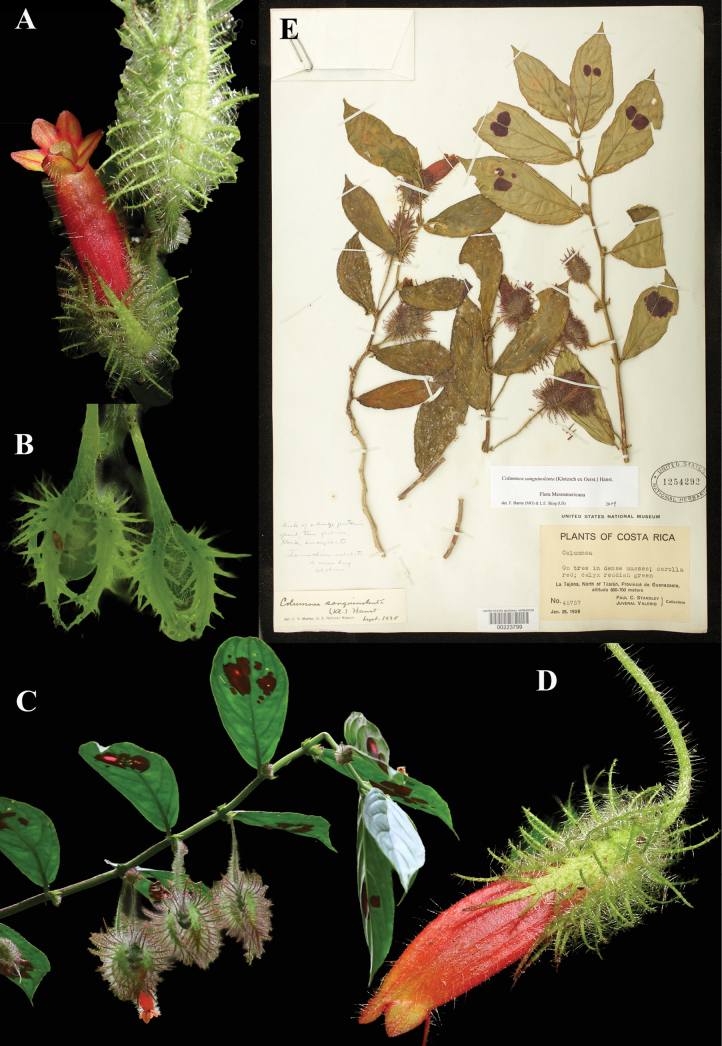
*Columnea sanguinolenta* (Klotzsch ex Oerst.) Hanst. A. Mature flower; B. Fruits; C. Habit; D. Mature flower; E. Neotype from US of *Standley & Valerio 45757* [US00223799] (A. iNaturalist observation 19720187; B. iNaturalist observation 100539758; C. iNaturalist observation 39758987; C. iNaturalist observation 209095419; D. iNaturalist observation 19720187). Photos (A, D) by Steven Daniel, (B) by Cindy Howland-Hodson, (C) by Clinton Dexter-Nienhaus.

**Comments.**Oersted (1858: 49) cites a *Warszewicz* specimen in the Berlin herbarium, as does Hanstein, who, in addition, cites a Wendland specimen, *Wendland 807*. Both are presumed destroyed and no duplicates have been located, thus a neotype has been designated (Fig. 25E).

*Columnea sanguinolenta* can be confused with *C. grata*, but *C. sanguinolenta* can be distinguished by having pedicels shorter than the leaves, and calyx fimbriae longer than 4 mm.

### ﻿*Columnea schiedeana* Schltdl., Linnaea 8: 249 (1833). Lectotype, designated here: Mexico, *Schiede 128* (HAL (image)! [HAL0082525]); isolectotypes: HAL (image)! (HAL0107384), MO! [MO acc. no. 212062], NY! [NY3312596], P (image)! [P00603449].

Fig. 26

**Figure 26.**
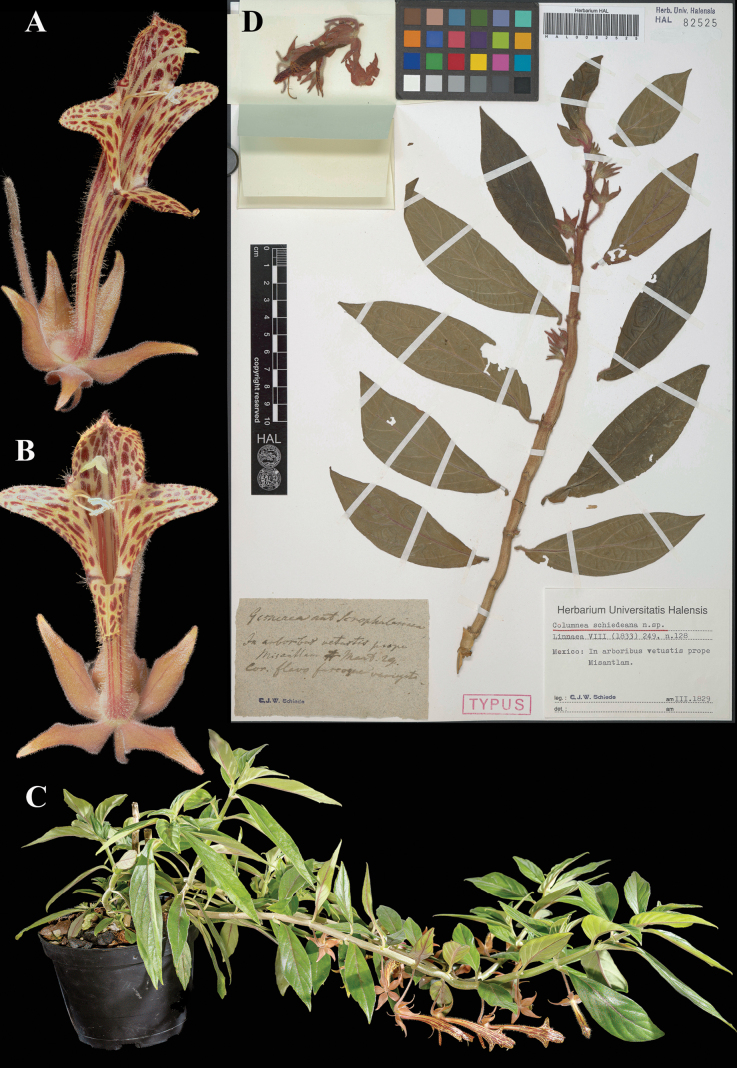
*Columnea schiedeana* Schltdl. A, B. Mature flowers; C. Habit; D. Lectotype from HAL of *Schiede 128* [HAL0082525] (A–C. from *J.L. Clark & W. Collier 18369*). Photos by Wade Collier, (E) Specimen image reproduced with permission from Martin-Luther-Universität-Halle-Wittenberg herbarium (HAL), Germany.

**Comments.** There are two specimens of this gathering at HAL, as well as duplicates at MO, NY, and P. We selected as lectotype the more complete specimen at HAL (Fig. 26D) where the main collection of gatherings used by Schlechtendal reside.

### ﻿*Columnea sulfurea* Donn. Sm., Bot. Gaz. 31: 117 (1901). Lectotype (first-step), designated by Gibson (1974: 270); second-step, designated here: Guatemala, *Tuerckheim 7646* (US!) [US00126505], isolectotypes (US!) [US00126506; US00126507].

Fig. 27

**Figure 27.**
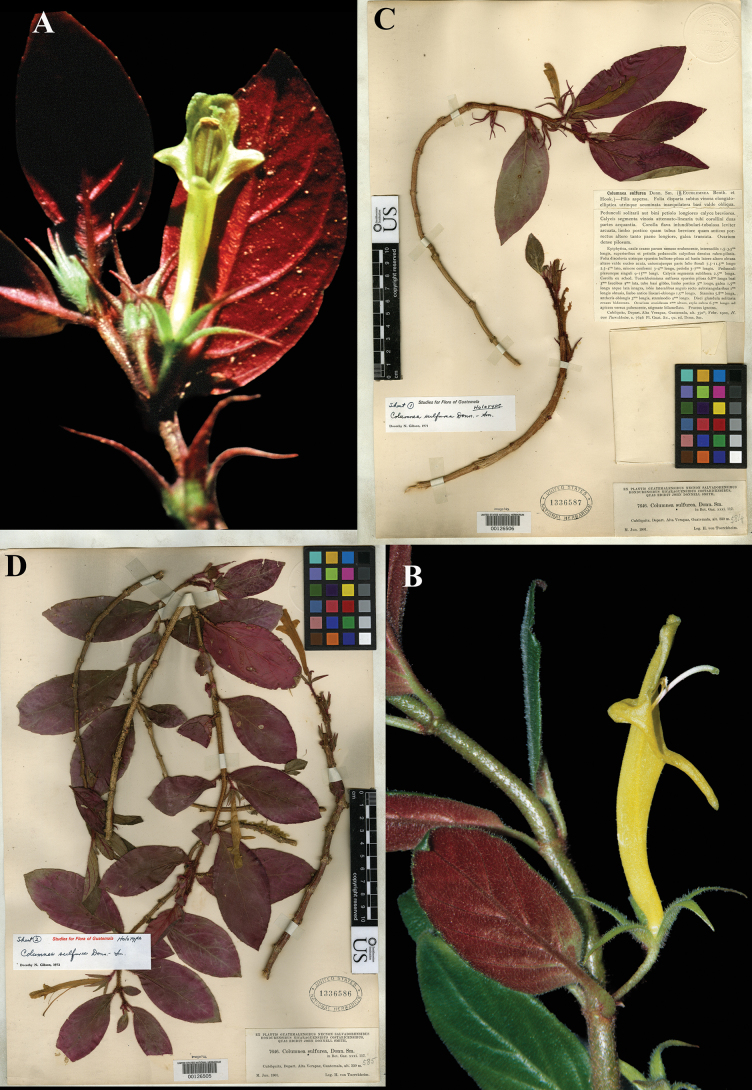
*Columnea sulfurea* Donn. Sm. A, B. Habit; C. isolectotype [‘sheet 1’ US00126506] D. Lectotype from US of *Tuerckheim 7646* [‘sheet 2’ US00126505] (A, B. *J.L. Clark 6275*). Photos (A, B) by John L. Clark.

**Comments.** There is a discrepancy between the collection date of the type cited in the protologue, February 1900, and the date on the type labels, May 1901 (later than the publication date, February 1901). We interpret the date on the labels (see Fig. 27 C, D) as a printing error. The dates on syntypes at GH and NY are the same. The same incorrect date appears on the labels of type specimens of other species as well (see *Drymonia macrantha* below).

Gibson labeled two Tuerckheim specimens as holotypes (‘sheet 1’ and sheet 2’). We have designated ‘sheet 2’, the more complete specimen (US00126505; Fig. 27D) as lectotype, and ‘sheet 1’ (US00126506; Fig. 27C) as an isolectotype.

### ﻿*Columnea tenuis* Klotzsch ex Oerst., Centralamer. Gesner. 63 (1858). Neotype, designated here: Panama, Chiriquí, Bajo Chorro, near Rio Caldera, 21 Mar 1977, *Skog 4071* (US! [US00223894].

Fig. 28

**Figure 28.**
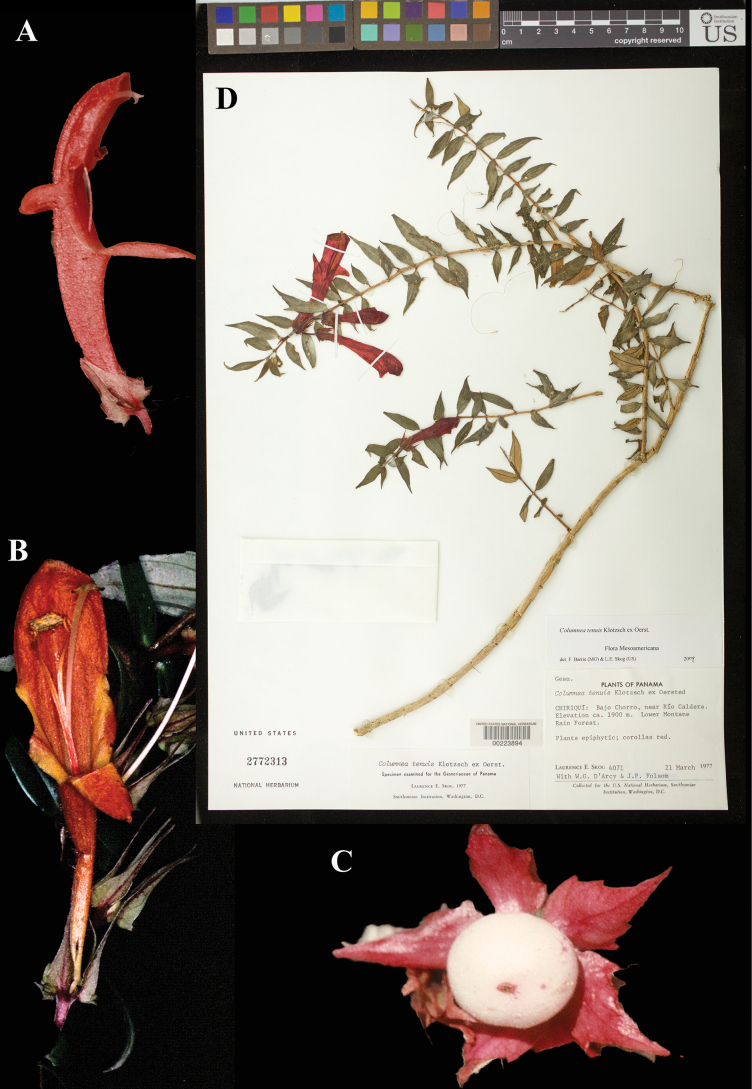
*Columnea tenuis* Klotzsch ex Oerst. A, B. Flowers; C. Fruit; D. Neotype from US of *Skog 4071* [US00223894] (A, C. *L.E. Skog 4052*; B. *R.W. Dunn 9907060*). Photos (A, C) by Laurence E. Skog, (B) by Richard W. Dunn.

**Comments.** Oersted cites a *Warszewicz* specimen representing this species, which would be the holotype, if extant. Hanstein (1865: 406) lists “*Warszewicz n. 38*; *n. 36* (*243*)?; *Wendland n. 547*” as this species. None of the collections cited by Oersted or Hanstein have been found as they were destroyed at B, and no duplicates have been located. Thus, a neotype (Fig. 28D) from Panama has been designated.

### ﻿*Drymonia alloplectoides* Hanst., Linnaea 34: 358 (1865). Neotype, designated here: Costa Rica, Puntarenas, Canton de Osa, Golfo Dulce area, 0 m, 6 Jun 1949, *Allen 5297* (US! [US00082232]).

Fig. 29

**Figure 29.**
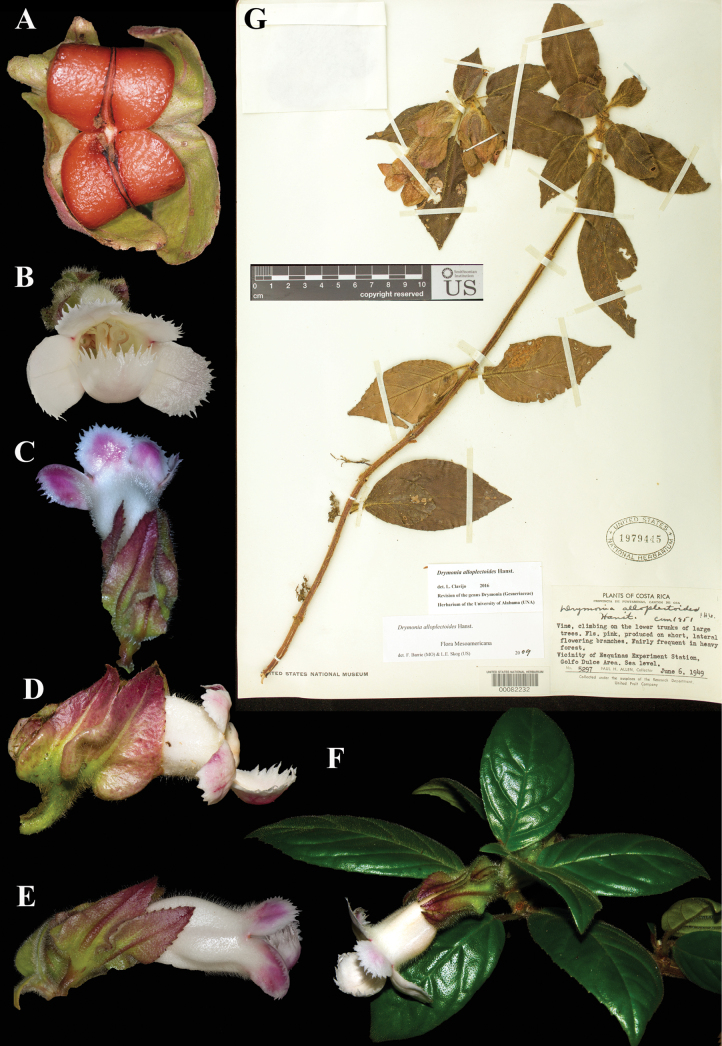
*Drymonia alloplectoides* Hanst. A. Mature bivalved fleshy capsule; B. Front view of corolla; C. Dorsal view of flower; D, E. Lateral view of flowers; F. Habit; G. Neotype from US of *Allen 5297* [US00082232] (A. *J.L. Clark 8268*; B. *J.L. Clark & W. Collier 18642*; C. *J.L. Clark 11313*; D. *J.L. Clark et al. 16612*; E. *J.L. Clark 11313*; F. *J.L. Clark & J. Katzenstein 13122*). Photos (A–F) by John L. Clark.

**Comments.***Drymonia alloplectoides* was described by Hanstein, based on *Warszewicz 34* at B, subsequently destroyed. Thus, a neotype from Costa Rica was selected (Fig. 29G).

### ﻿*Drymonia conchocalyx* Hanst., Linnaea 34: 360 (1865). Lectotype, designated here: Costa Rica, *Wendland 954*] (GOET (image)! [GOET003910]).

Fig. 30

**Figure 30.**
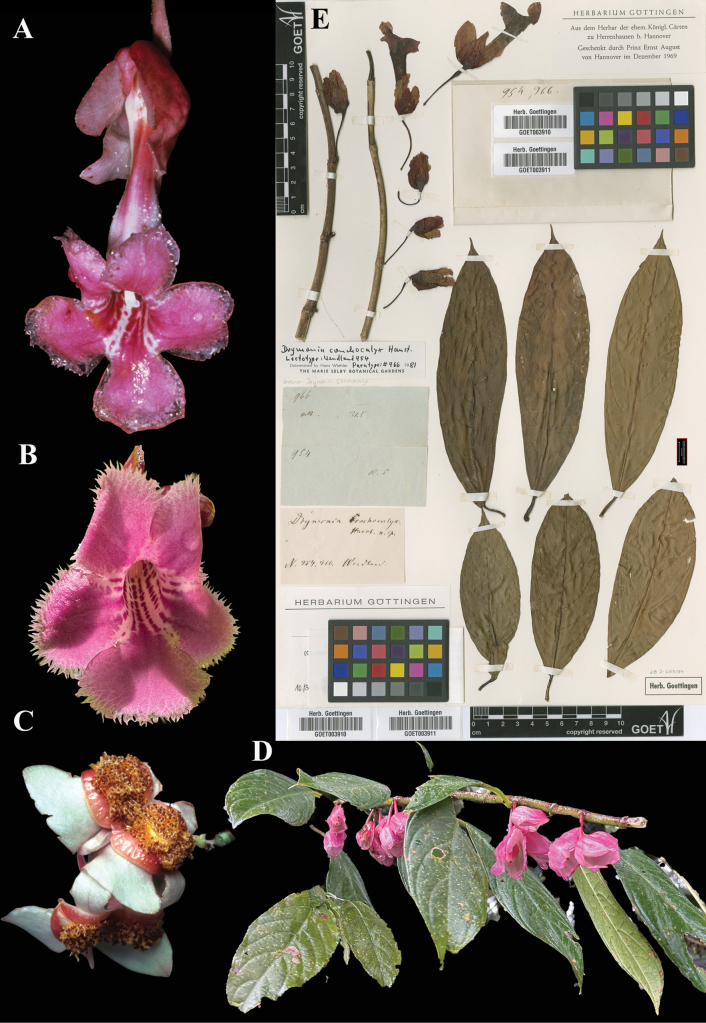
*Drymonia conchocalyx* Hanst. A. Dorsal view of mature flower; B. Front view of corolla; C. Mature bivalved capsule; D. Habit; E. Lectotype from GOET of *Wendland 954* [GOET003910] (A. *Robbin Moran sn*; B. iNaturalist observation 275269525; C. *H. Wiehler 2687*; D. iNaturalist observation 256223790). Photos (A) by Robbin Moran, (B) by D. Alexander Carillo-Martínez, (C) by Hans Wiehler, (D) by David Rankin, (E) Specimen image reproduced with permission from the herbarium at the Universität Göttingen (GOET), Germany.

**Comments.***Drymonia conchocalyx* was described by Hanstein based on *Wendland 954* and *966* at B, specimens subsequently destroyed. Dowe et al. (2022: 52–53) wrote that Morton (1938: 1172) lectotypified this species. Morton cited *Wendland 954* but, as noted above, Morton (1938) did not state an intention to designate types. The GOET specimen of *Wendland 954* is mounted on the same sheet as *Wendland 966* (see Fig. 30E), and it is not possible to discern which specimen corresponds to which collection number. Each has been given a different barcode, however, and, because the specimens are virtually identical, there is no confusion as to the application of the name, regardless of which specimen is considered to be *Wendland 954* and which is the syntype, *Wendland 966*.

### ﻿*Drymonia macrantha* (Donn. Sm.) D.N.Gibson, Phytologia 23: 336 (1972). *Alloplectus macranthus* Donn. Sm., Bot. Gaz. 31: 117 (1901). Lectotype (first-step), designated by Gibson (1974: 275); second-step, designated here: Guatemala, *Tuerckheim 7642* (US!) [US00126435].

Fig. 31

**Figure 31.**
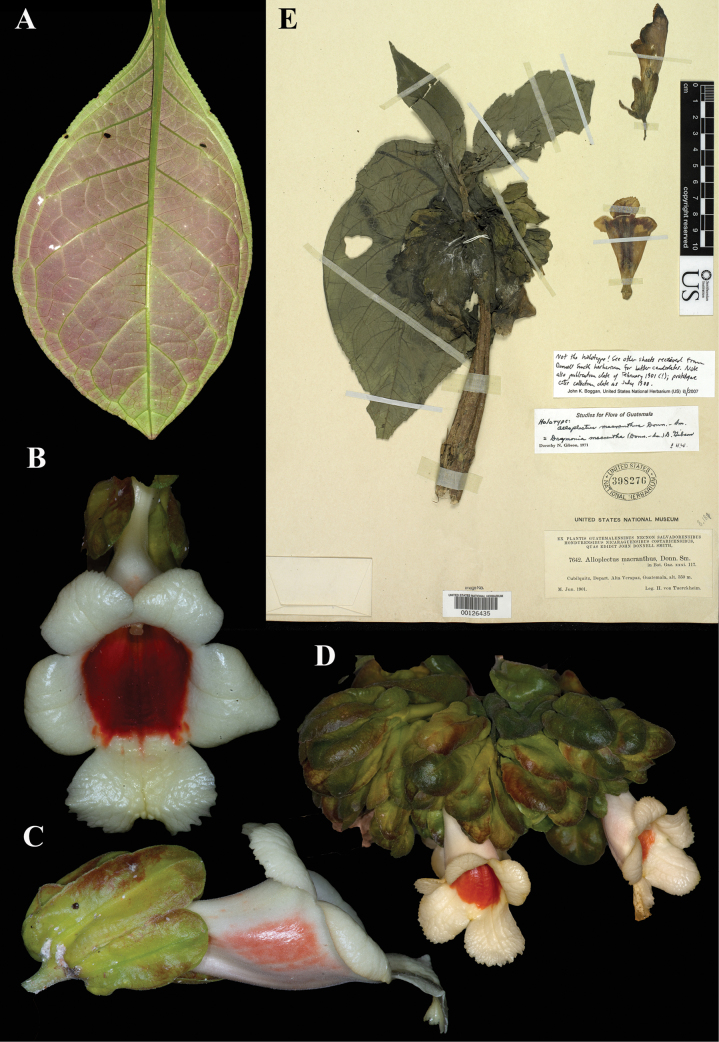
*Drymonia macrantha* (Donn. Sm.) D.N. Gibson. A. Abaxial leaf surface; B. Front view of corolla; C. Lateral view of flower; D. Inflorescence; E. Lectotype from US of *Tuerckheim 7642* [US00126435] (A–C. *J.L. Clark 16373*; D. *J.L. Clark 11314*). Photos (A–D) by John L. Clark.

**Comments.** The four sheets of this number at US, as well as duplicates at A, GH, K, M, and NY, all bear the date, June 1901. This is after the publication date of the protologue, February 1901. The protologue gives the specimen collection date as July 1900. We interpret the date (see Fig. 31E) on the label as a printing error, similar to that on the labels for *Columnea sulfurea* (Fig. 27 C, D).

### ﻿*Drymonia oinochrophylla* (Donn. Sm.) D.N.Gibson, Phytologia 23: 336 (1972), *Alloplectus oinochrophyllus* Donn. Sm., Bot. Gaz. 54: 239 (1912). Lectotype (first-step) Gibson (1972: 336); second-step, designated here: Guatemala, Pansamalá, *Tuerckheim 1080* (US! [US00126434]).

Fig. 32

**Figure 32.**
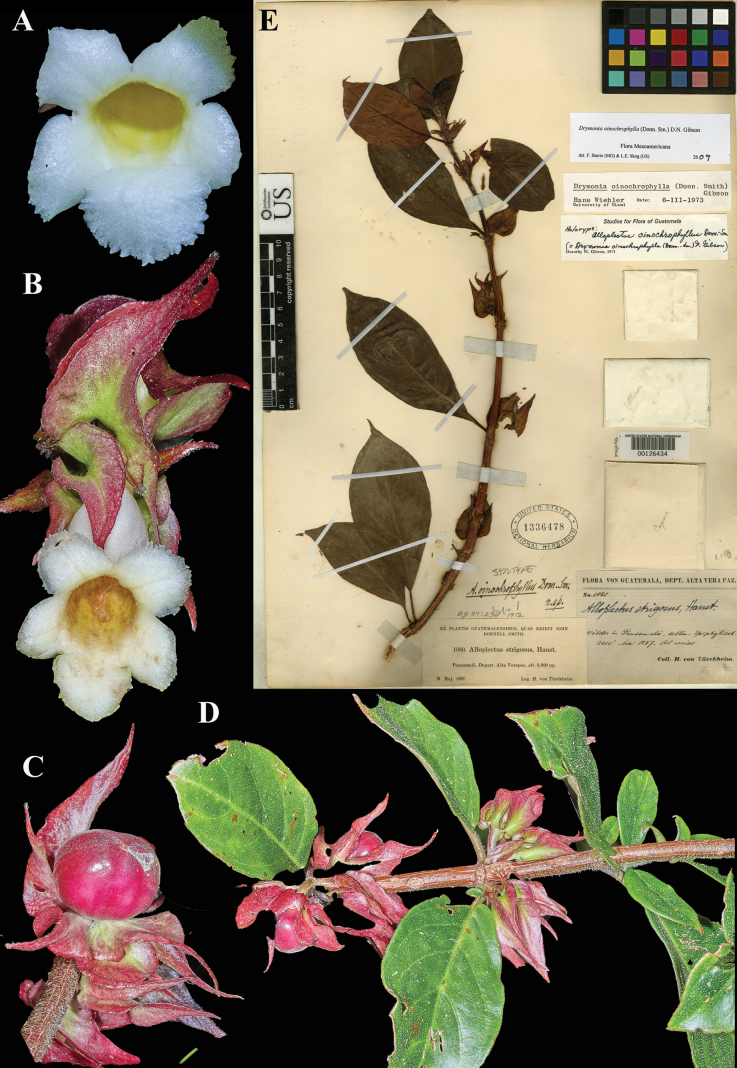
*Drymonia oinochrophylla* (Donn. Sm.) D.N. Gibson. A. Front view of corolla; B. Mature flower; C. Mature globose fruit; D. Habit; E. Lectotype from US of *Tuerckheim 1080* [US00126434] (A. iNaturalist observation 110297394; B–D. iNaturalist observation 103973673). Photos (A–D) by Sune Holt.

**Comments.**Gibson (1972) provided the first step in the lectotypification of *Alloplectus oinochrophyllus* when she selected *Tuerckheim 1080* from among the syntypes listed in the original Donnell Smith protologue and annotated one of them as holotype (see Fig. 32E). However, Gibson did not specify the herbarium location of the specimen in either her *Phytologia* (Gibson 1972) article or in the “Flora of Guatemala” (Gibson 1974). We provide the second-step lectotypification here.

### ﻿*Drymonia ovata* Hanst., Linnaea 34: 355. (1865). Lectotype, designated here: Costa Rica, *Hoffmann 545* (GOET (image)! [GOET003888]).

Fig. 33

**Figure 33.**
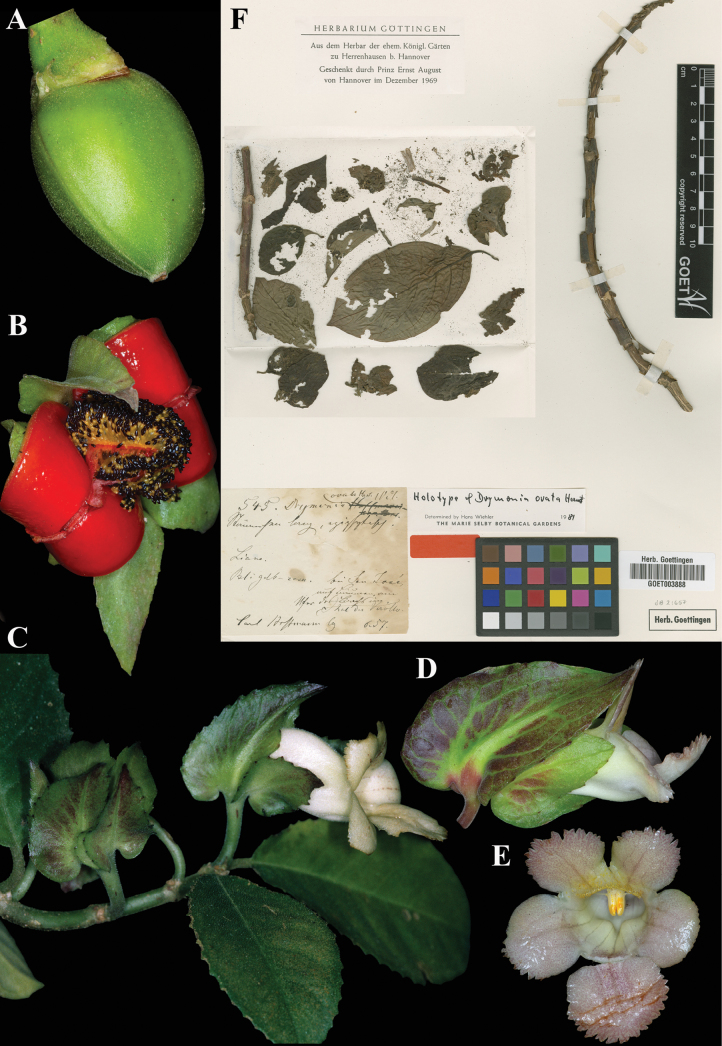
*Drymonia ovata* Hanst. A. Immature fruit; B. Mature fruit; C. Habit; D. Lateral view of flower; E. Front view of corolla; F. Lectotype from GOET of *Hoffmann 545* [GOET003888] (A. *J.L. Clark et al. 12889*; B. *J.L. Clark et al. 19649*; C. *J.L. Clark & A. Fernandez 6896*; D, E. *J.L. Clark et al. 19649*). Photos (A–E) by John L. Clark, (E) Specimen image reproduced with permission from the herbarium at the Universität Göttingen (GOET), Germany.

**Comments.** The holotype was at B, as noted by Hanstein in the original publication, but subsequently destroyed. The lectotype (Fig. 33F) is the only known extant specimen of *Hoffmann 545*. It was annotated as holotype by Wiehler, a determination he did not publish.

This name is a synonym of the wide-spread species, *Drymonia serrulata* (Jacq.) Mart.

### ﻿*Drymonia parviflora* Hanst., Linnaea 34: 352 (1865). Neotype, designated here: Costa Rica, *Davidse 28369* (MO! [acc. no. 3212186]); isoneotype (US! [US 00992675]).

Fig. 34

**Figure 34.**
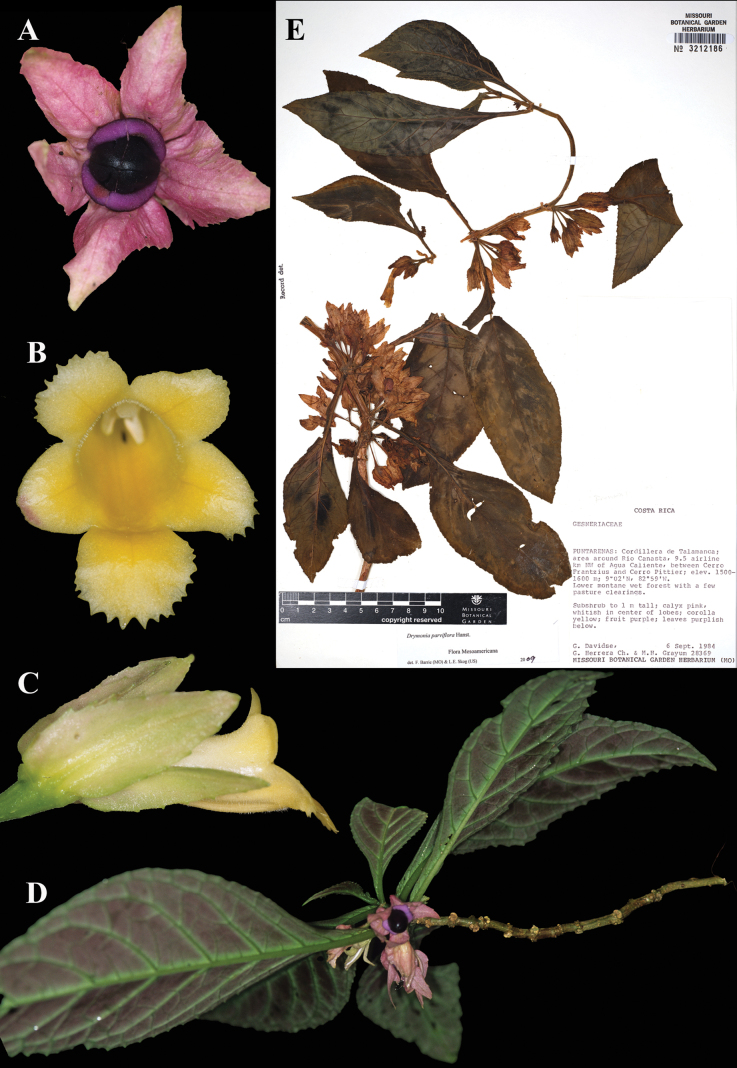
*Drymonia parviflora* Hanst. A. Mature fruit; B. Front view of corolla; C. Lateral view of flower; D. Habit; E. Lectotype from MO of *Davidse 28369* [acc. no. 3212186] (A–C. *J.L. Clark & J. Katzenstein 8676*; D. *J.L. Clark & C. Espinosa 8601*). Photos (A–D) by John L. Clark, (E) Specimen image reproduced with permission from the Missouri Botanical Garden (MO).

**Comments.** Hanstein based this species on a specimen from Costa Rica, *Hoffmann 798*, at Berlin (B), which is no longer extant. No duplicates of the type have been found, thus a neotype is designated here. Very few specimens of this species bear complete flowers, thus we selected one that does (see Fig. 34E).

### ﻿*Drymonia turrialvae* Hanst., Linnaea 34: 359–360 (1865). Holotype: Costa Rica, *Wendland 517* (B, destroyed); lectotype, designated here: Costa Rica, “Vulc. de Turrialba”, *Wendland 517* (GOET (image)! [GOET003890]).

Fig. 35

**Figure 35.**
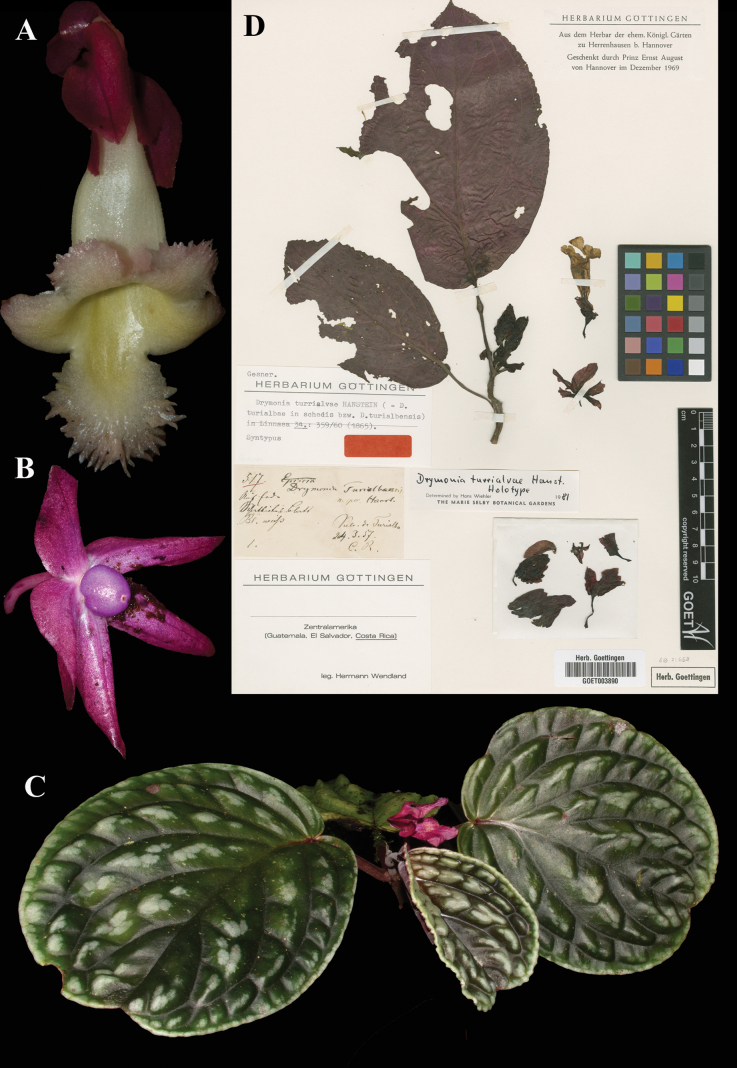
*Drymonia turrialvae* Hanst. A. Dorsal view of mature flower; B. Fruit; C. Habit; D. Lectotype from GOET of *Wendland 517* [GOET003890] (A, B. *J.L. Clark & I. Pizarro 12628*; C. *J.L. Clark et al. 19285*). Photos (A–E) by John L. Clark, (D) Specimen image reproduced with permission from the herbarium at the Universität Göttingen (GOET), Germany.

**Comments.**Dowe et al. (2022: 53) wrote that Leeuwenberg (1958: 398) lectotypified this species, but Leeuwenberg cited *Wendland 517* (B) as type, noting that it had been destroyed, and did not mention the GOET duplicate. Wiehler annotated the GOET specimen as holotype (see Fig. 35D) but did not publish his determination.

### ﻿*Gasteranthus wendlandianus* (Hanst.) Wiehler, Selbyana 1: 156 (1977). *Besleria wendlandiana* Hanst., Linnaea 34: 318 (1865). Lectotype, designated by Skog & Kvist, Syst. Bot. Monog. 59: 102 (2000): Costa Rica, *Wendland 922* (GOET (image)! [GOET003896]).

Fig. 36

**Figure 36.**
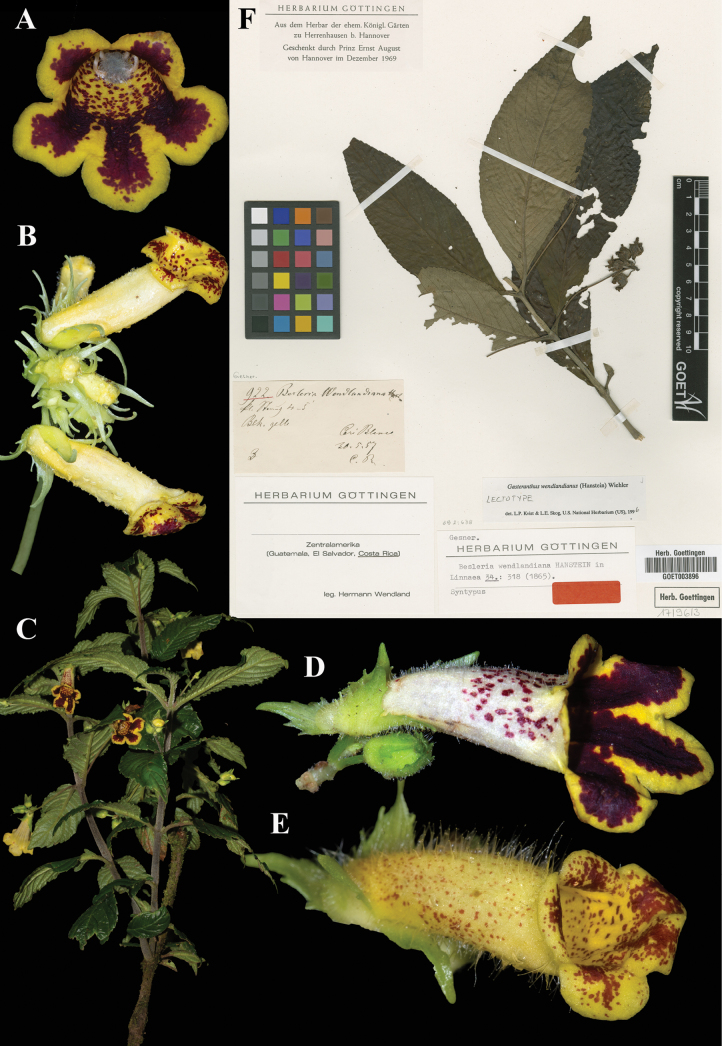
*Gasteranthus wendlandianus* (Hanst.) Wiehler. A. Front view of corolla; B. Inflorescence; C. Habit; D, E. Lateral views of mature flowers; F. Lectotype from GOET of *Wendland 922* [GOET003896] (A. *J.L. Clark et al. 18070*; B. *J.L. Clark et al. 19067*; C, D. *J.L. Clark et al. 18070*; E. *J.L. Clark et al. 11778*). Photos (A–E) by John L. Clark, (F) Specimen image reproduced with permission from the herbarium at the Universität Göttingen (GOET), Germany.

**Comments.** Hanstein listed two Wendland specimens for this species, 568 and 922. Morton (1938: 1156) mentioned only *Wendland 568*, probably as that was the first species listed by Hanstein in the protologue. However, in his revision of *Besleria* in 1939, Morton cited *Wendland 922* at B [since destroyed] as the “type,” correctable under the current Code to lectotype. Skog and Kvist (2000: 102) designated the GOET specimen (Fig. 36F) as the replacement lectotype. Dowe et al. (2022: 52) appear to credit Morton with selecting the GOET specimen as the lectotype, but there is no mention of GOET in the 1939 revision pertaining to this species.

### ﻿*Moussonia deppeana* (Schltdl. & Cham.) Hanst., Linnaea 34: 284 (1865). *Gesneria deppeana* Schltdl. & Cham., Linnaea 5: 110. (1830). Lectotype: Mexico, *Schiede 186* (first-step), designated by Ramírez-Roa and Chiang (2010: 299); second-step (designated here): Mexico, *Schiede 186* (HAL (image)! [HAL 096235]); isolectotype (HAL (image)! [HAL 0114003].

Fig. 37

**Figure 37.**
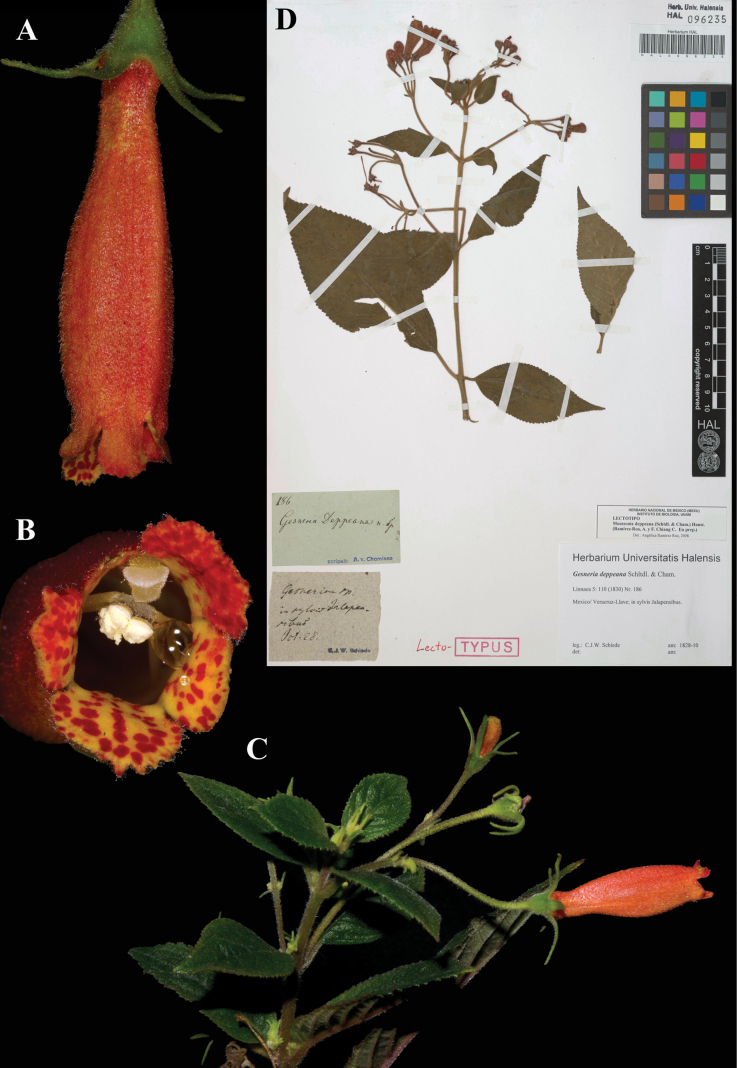
*Moussonia deppeana* (Schltdl. & Cham.) Hanst. A. Lateral view of mature flower; B. Front view of corolla; C. Habit; D. Lectotype from HAL of *Schiede 186* [HAL096235] (A–C. *J.L. Clark et al. 13108*). Photos (A–C) by John L. Clark, (D) Specimen image reproduced with permission from Martin-Luther-Universität-Halle-Wittenberg herbarium (HAL), Germany

**Comments.** In their proposal to conserve the name *Moussonia* with a conserved type, Ramírez-Roa and Chiang (2010: 298–299) designated “*Schiede 186* (HAL)”, as “lectotype”. However, there are two specimens of this number at HAL, and Ramírez-Roa & Chiang did not specify which specimen was the lectotype. We select the specimen with the barcode 096235, labeled ‘TYPUS’, as the lectotype (Fig. 37D); the other specimen, HAL114003, is labeled ‘isolectotypus’. This Schiede type specimen collection number also appears on other specimens of other species in other plant families according to the images in JStorPlants (www.plants.jstor.org).

### ﻿*Niphaea oblonga* Lindl., Bot. Reg. 27: Misc. 80 (1841). Neotype, designated here: Cultivated in London from material collected in Guatemala by Hartweg, *Cult. Hort. Soc. London s.n.* (K (image!) [K000479996]).

Fig. 38

**Figure 38.**
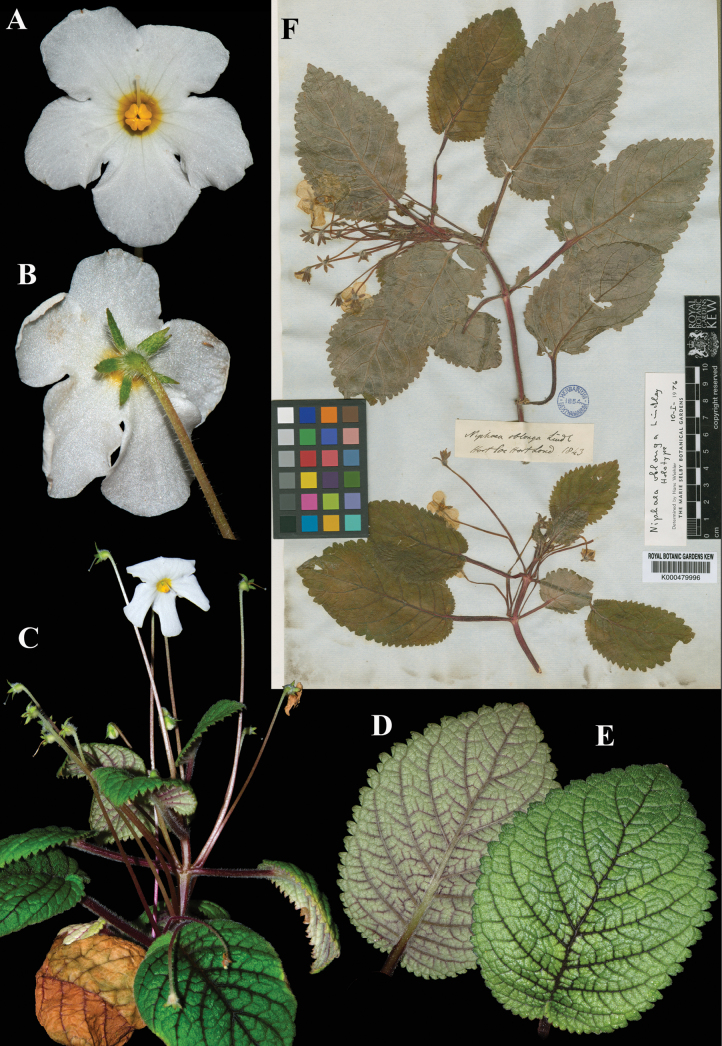
*Niphaea oblonga* Lindl. A. Front view of corolla; B. Calyx; C. Habit; D. Abaxial leaf surface; E. Adaxial leaf surface; F. Neotype from K of *Cult. Hort. Soc. London s.n.* [K000479996] (A, B. *J.L. Clark 15986*; C. *J.L. Clark et al. 14539*; D, E. *J.L. Clark 15986*). Photos (A–E) by John L. Clark, (F) Specimen image reproduced with permission from the Board of Trustees of the RBG, Kew.

**Comments.** The specimen at Kew was most likely made from the material that Hartweg brought back from Guatemala. It was annotated by Wiehler as “holotype” (see Fig. 38F), but there is no evidence that he published this typification. The earliest mention of the *Cult. Hort. Soc. London s.n.* specimen at Kew being the type was in Boggan et al. (2008, p. 173), however, the authors did not use the terms “designated here”, required after 2001 (Turland et al. 2025, Art. 7.11), to tie down the typification. The specimen cannot be the holotype as it is dated 1843, two years after the publication of the name by Lindley, but it is an appropriate choice for neotype.

### ﻿*Rufodorsia congestiflora* (Donn. Sm.) Wiehler. Selbyana 1: 146 (1973). *Besleria congestiflora* Donn. Sm., Bot. Gaz. 61: 379 (1916). Lectotype, designated by Wiehler (1975, p. 147): Costa Rica, *Tonduz 12658* (US! [US00126091]).

Fig. 39

**Figure 39.**
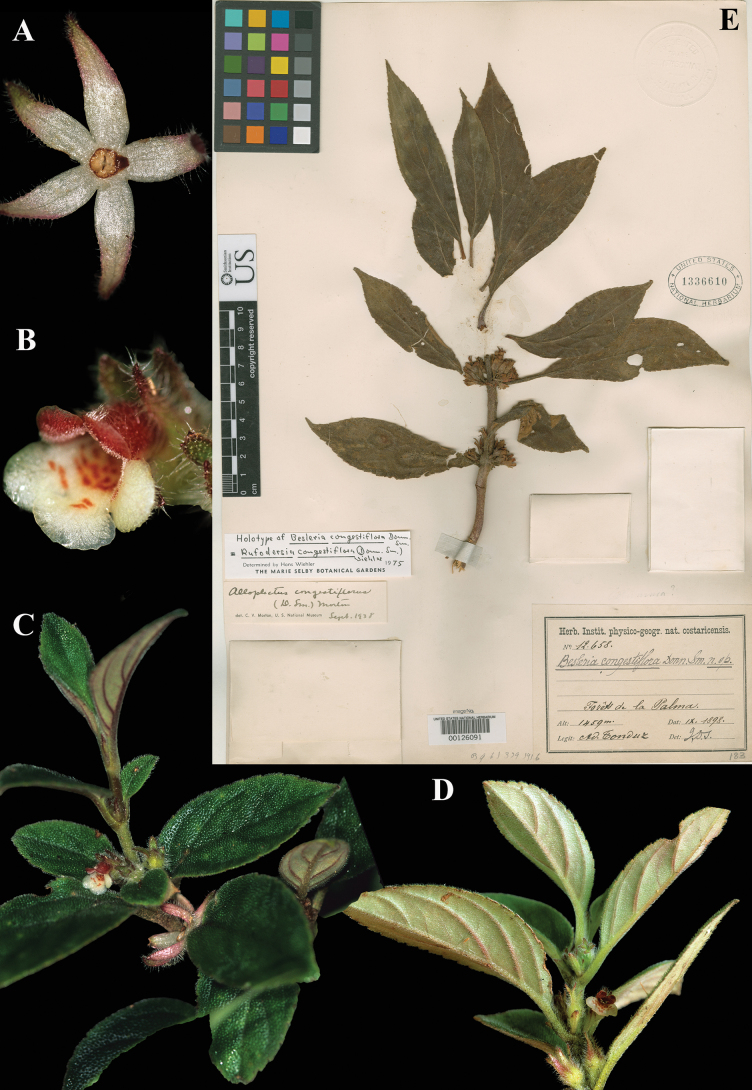
*Rufodorsia congestiflora* (Donn. Sm.) Wiehler. A. Calyx with nectary; B. Flower; C, D. Habit; E. Lectotype from US of *Tonduz 12658* [US00126091] (A–D. *J.L. Clark & L. Espinosa 8577*). Photos (A–D) by John L. Clark.

**Comments.** Wiehler annotated the two specimens of *Tonduz 12658* at US as holotype (see Fig. 39E) and isotype, thus typifying and tying names to the specimens at US. Although Wiehler did not identify in print which specimen was the holotype, the fact that he annotated the specimen as holotype, the specimen can be linked and therefore his statement that the specimen was the holotype may be corrected to lectotype.

### ﻿*Rhynchoglossum azureum* (Schltdl.) B.L. Burtt, Notes Roy. Bot. Gard. Edinburgh 24: 168 (1962). *Klugia azurea* Schltdl., Linnaea 8: 248 (1833). Lectotype, designated here: Mexico, *Schiede 127* (HAL (image)! [HAL098436]), isolectotype (NY (image)! [NY00312969]).

Fig. 40

**Figure 40.**
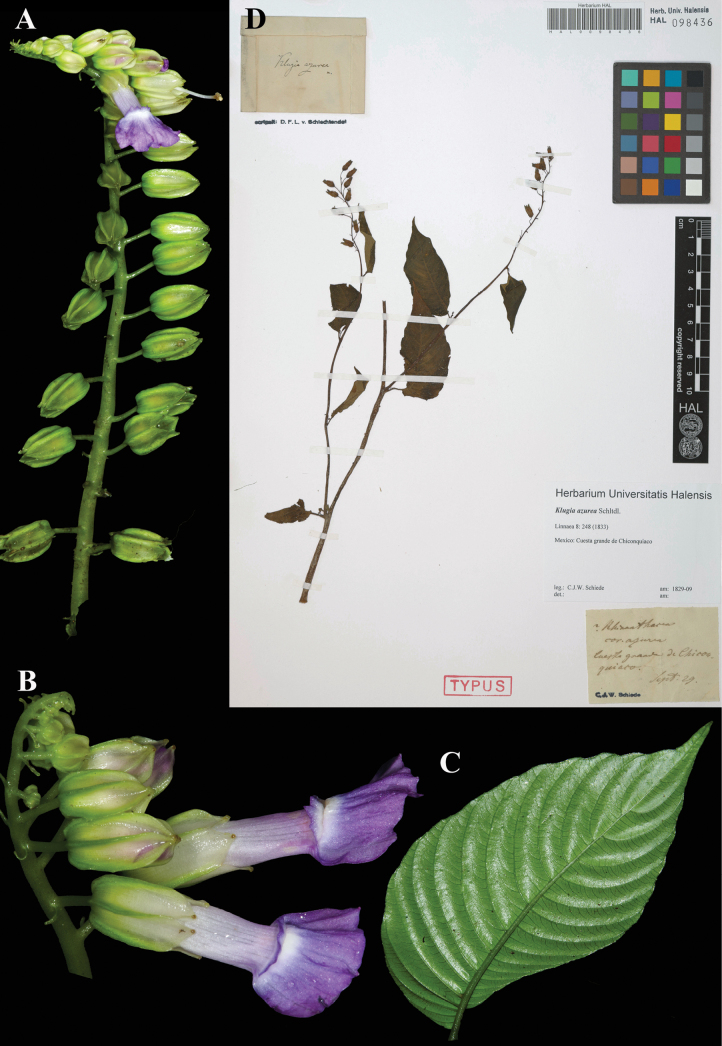
*Rynchoglossum azureum* (Schltdl.) B.L. Burtt. A. Inflorescence; B. Flowers; C. Abaxial leaf surface; D. Lectotype from HAL of *Schiede 127* [HAL098436] (A. *J.L. Clark et al. 13831*; B. *J.L. Clark 13905*; C. *J.L. Clark et al. 13224*). Photos (A–C) by John L. Clark, (D) Specimen image reproduced with the permission from the Martin-Luther-Universität-Halle-Wittemberg herbarium (HAL), Germany.

**Comments.** There are 3 specimens of *Schiede 127* at HAL, but only the one with the date cited in the protologue, September 1829, is designated as lectotype (Fig. 40D). The specimen at NY also has the same date, as does the specimen at MO. However, the date on the MO specimen is in a different handwriting and may have been taken from the protologue and is therefore not reliable.

### ﻿*Smithiantha cinnabarina* (Linden) Kuntze, Revis. Gen. Pl. 2: 978 (1891). Gesneria (Naegelia) cinnabarina Linden, Hort. Lind. (Catalogue des Plantes Exotiques) 12: 2–3 (1857). Lectotype, designated here: Illustration in J. J. Linden, *Hort. Lind.* 12 (1857): unnumbered page and plate “*GesneriaNaegeliaCinnabarina*, Lind.”

Fig. 41

**Figure 41.**
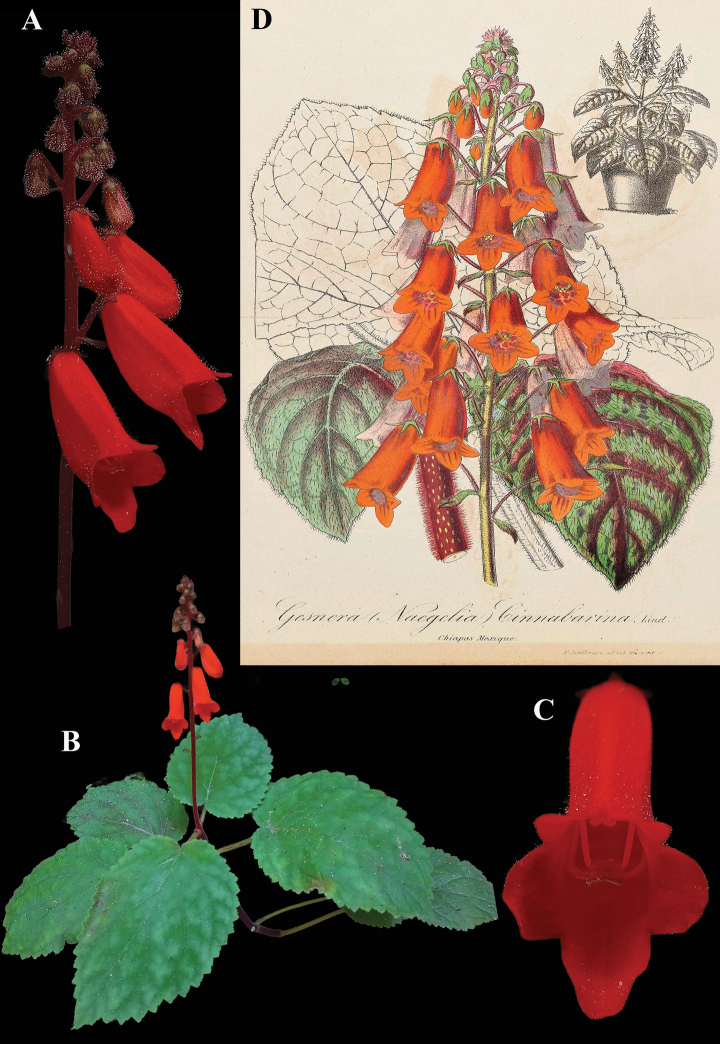
*Smithiantha cinnabarina* (Linden) Kuntze. A. Inflorescence; B. Habit; C. Dorsal view of flower; D. Lectotype of plate from Linden (1857) (A–C. from iNaturalist observation 105587887). Photos (A–C) by Saul Miguel-Z.

**Comments.** According to the protologue, the plants originated in Chiapas, Mexico, were collected by Ghiesbreght and brought into cultivation. The plate in *Hort. Lind*. (Fig. 41D) is original material as it was published at the same time as the description and is part of the protologue.

### ﻿*Solenophora calycosa* Donn. Sm., Bot. Gaz. 25: 152 (1898). Lectotype, designated by Skog (1979: 987), as “type”: Costa Rica, *Pittier 283* (CR (image!) [cat. no. CR283], photos F!, US!).

Fig. 42

**Figure 42.**
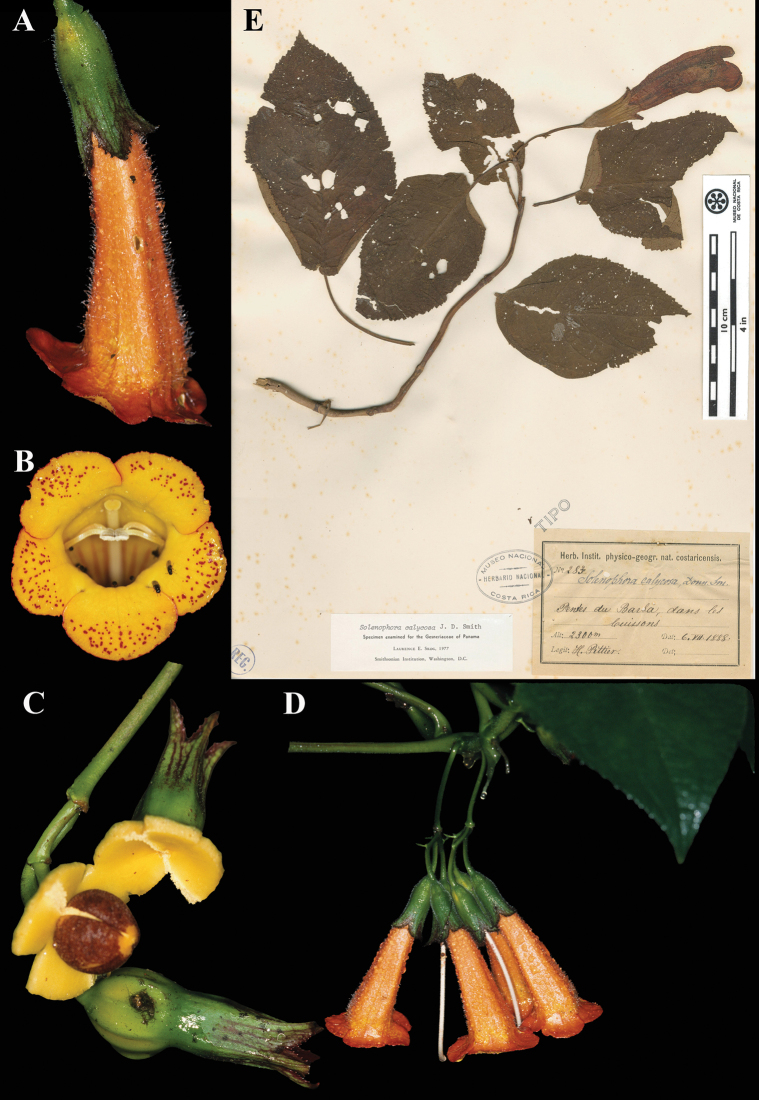
*Solenophora calycosa* Donn. Sm. A. Mature flower; B. Front view of corolla; C. Mature fruit; D. Inflorescence; E. Lectotype from CR of *Pittier 283* [cat. no. CR283] (A, B. *J.L. Clark 8715*; C. *J.L. Clark 8726*; D. *J.L. Clark 8715*). Photos (A–D) by John L. Clark, (E) Specimen image reproduced with the permission from the Museo Nacional de Costa Rica (CR)

**Comments.** John Donnell Smith in the protologue published in 1898 cited two collections for this species, *Pittier 283* and *Tonduz 2022*, thus syntypes, and that they were at “herb. Nat. C. R.”. Although Donnell Smith transferred many of his cited type specimens to US, he did not transfer the *Pittier 283* specimen (Fig. 42E). There is a *Pittier 2022* specimen from the Donnell Smith herbarium at US, and this is likely a duplicate of *Tonduz 2022* as it has the same locality and date of the Tonduz collection cited by Donnell Smith. Skog (1979) in the treatment of Gesneriaceae for the Flora of Panama appears to be the first to indicate *Pittier 283* (CR) as “Type”, a statement that effectively lectotypifies *Solenophora calycosa*. Weigend and Förther (2002) repeated the citation of the Pittier specimen as “Type”.

## ﻿Additional information

### Use of AI

No use of AI was reported.

### Author ORCIDs

Fred R. Barrie https://orcid.org/0000-0002-1532-3609

Laurence E. Skog https://orcid.org/0000-0003-3655-7516

John L. Clark https://orcid.org/0000-0002-1414-6380

Kimberly Hansen https://orcid.org/0009-0007-3956-8399

### Data availability

All of the data that support the findings of this study are available in the main text.
